# A deep learning-based evaluation system for child-friendly urban streets integrating abstract and concrete features—A case of Shanghai Urban Street

**DOI:** 10.1371/journal.pone.0342430

**Published:** 2026-02-23

**Authors:** Huijun Tu, Xudong Miao, Shitao Jin

**Affiliations:** 1 College of Architecture and Urban Planning, Tongji University, Shanghai, China; 2 Shanghai Key Laboratory of Urban Renewal and Spatial Optimization Technology, Shanghai, China; Zhejiang Agriculture and Forestry University: Zhejiang A and F University, CHINA

## Abstract

To address the challenges of high subjectivity, difficult data acquisition, and low efficiency in current evaluation methods for child-friendly urban streets, this study proposes a deep learning-based evaluation system that integrates both concrete and abstract features. The study utilizes 1,322 street samples in Shanghai, integrating 50 quantifiable concrete features with abstract features extracted from 6,724 street view images. Perceptual survey data from children aged 7–12 were incorporated as the target output for model training. Methodologically, abstract features were first extracted from streetscapes using a ResNet18 convolutional neural network. These features were then fused with the concrete features, and a multi-layer artificial neural network was constructed to predict child-friendliness. The results demonstrate that the model achieved an average accuracy of 96.91% on the validation set and an overall accuracy of 97.35% on the test set, indicating its effectiveness in identifying street samples with low levels of child-friendliness. Further case validation demonstrates the model’s capability to rapidly identify child-unfriendly spatial characteristics at an urban scale, including poor traffic safety, inadequate pedestrian environments, and a lack of engaging elements. This study offers a novel technical pathway for the quantitative evaluation and targeted management of child-friendly streets.

## 1. Introduction

The global advancement of the “Child-Friendly Cities” initiative has positioned the environmental quality of urban spaces as a critical benchmark for evaluating urban governance and public service capabilities, particularly in terms of children’s survival and development [1]. Streets, as the most fundamental and ubiquitous public space units in cities, serve as primary venues for children’s daily mobility, recreation, and social interactions. Their environmental quality directly influences children’s sense of safety, belonging, and engagement [2]. However, under long-standing vehicle-centric and efficiency-oriented urban planning paradigms, children’s mobility needs and spatial rights have often been marginalized [3]. Pervasive issues—such as oversized street dimensions, complex traffic flows, inadequate public amenities, and visual barriers—expose children to safety risks and social exclusion in street environments, hindering their healthy development [4].

Current research on child-friendliness in urban streets is gradually increasing, with the primary methods still focusing on constructing evaluation indicator systems, collecting field data, conducting expert assessments or public questionnaires, and employing tools such as fuzzy comprehensive evaluation and analytic hierarchy process to score street spaces comprehensively [5]. These studies provide preliminary theoretical and technical support for building child-friendly cities; however, they generally suffer from several issues. First, the evaluation methods heavily rely on manual involvement, making data collection and processing time-consuming and labor-intensive, which struggles to meet the demands of rapid urban renewal and dynamic governance [6]. Second, the construction of evaluation indicators exhibits significant subjectivity, with weight allocation lacking objective justification and comparability across different regions [7]. Third, the collection of urban street features is often incomplete, focusing primarily on quantifiable characteristics. As cities expand and data accessibility improves, traditional methods fall short in meeting the needs of real-time assessment and large-scale analysis [8]. These limitations highlight the necessity of developing an automated, objective, and efficient tool for assessing child-friendliness.

Recent advances in deep learning present transformative opportunities for urban environmental perception [9]. These technologies enable automated feature extraction from objective street data and data-driven modeling for spatial analysis. Crucially, urban street feature data comprises the following two types:

(1) Concrete features: Quantifiable metrics (e.g., sidewalk dimensions, traffic volumes, green coverage ratios, building heights, amenity density) directly trainable in fully connected layers.
(2) Abstract features: Non-quantifiable attributes (e.g., landscape aesthetics, architectural styles, streetscape ambiance) captured via photos or videos. Given the computational intensity of video feature extraction, this study adopts street view imagery for efficiency. Abstract features must first undergo visual feature extraction via convolutional neural networks (CNNs) before fusion with concrete features for joint training.

The key focus of this study’s deep learning-based evaluation model is to integrate concrete and abstract urban street features for extracting comprehensive street feature data, thereby establishing a novel pathway for quantifying child-friendliness perception in urban streets. This approach leverages deep learning technologies to autonomously extract child-friendly semantic information from multi-source urban street data. It not only minimizes human intervention and enhances assessment efficiency but also enables comprehensive coverage across diverse urban environments, facilitating real-time monitoring of dynamic changes in children’s perception of street-level child-friendliness.

It is noteworthy that although the data samples for the models constructed in this study are all from the urban area of Shanghai, which imposes certain sample limitations, transfer learning methods can still be applied to adapt some of the model parameters for training child-friendliness evaluation models of urban street samples with different cultural backgrounds or involving different child populations, so as to meet the perceptual needs of diverse child groups across various urban streets.

The chapter structure of this paper is as follows: Chapter 2 presents a literature review, examining domestic and international research on child-friendliness in urban streets and the application progress of artificial intelligence in urban spatial analysis, while summarizing the characteristics and limitations of current research. Chapter 3 proposes the overall framework and design logic of the child-friendliness evaluation system for urban streets. Chapter 4 details the construction method of the automatic evaluation model, including data collection, model design, and training procedures. Chapter 5 introduces the application of transfer learning techniques and validates the model’s generalization capability across different cities and child populations. Chapter 6 conducts empirical analysis on typical urban blocks to verify the effectiveness and adaptability of the system. The final two chapters discuss the research findings and contributions, and propose future research directions.

## 2. Literature review

### 2.1 Research on child-friendly urban streets

Child-Friendly Cities encompass multiple dimensions, including public facilities, service systems, traffic safety, and educational environments [10]. With the growing international emphasis on protecting children’s rights, urban design has increasingly begun to incorporate the everyday experiences of children, particularly by providing more inclusive support in domains such as mobility, play, and spatial participation [4]. Streets, as critical arenas for children’s routines, have become key indicators of urban rights fulfillment [11]. Implementation varies globally: developed nations like Germany and the Netherlands emphasize pedestrian infrastructure and traffic-calming measures [12], while China integrates child well-being into “15-minute living circles” to shift toward human-centered spatial design [13].

Research on urban streets and child-friendliness has evolved from conceptual discourse to empirical evaluation, with increasingly diverse thematic focuses. These studies can be broadly categorized into the following areas: First, studies focusing on the impact of street environments on children’s travel behavior and health, such as analyzing the influence of street safety, green coverage, and traffic facility configurations on children’s walking preferences [14]. Second, research examining children’s subjective perceptions and preferences in street spaces, including the collection of satisfaction ratings and evaluations from children or caregivers through questionnaires, interviews, and participatory mapping, reveals the relationship between spatial elements and children’s psychological perceptions [15]. Third, investigations combining spatial planning and social policy perspectives to explore how institutional design and spatial governance mechanisms can promote the development of child-friendly street systems, such as traffic-calming measures, participatory design practices involving children, and the establishment of multi-stakeholder collaboration platforms [16]. Additionally, some studies have begun to situate children’s street experiences within broader sociocultural contexts, emphasizing the relational networks of children within families and communities, as well as the collective and intergenerational characteristics of their spatial use [17]. Recent research, exemplified by Sheng et al. [18], has attempted to quantify children’s visual focus points using eye-tracking technology, providing an in-depth understanding of children’s preferences for streetscape elements from a perceptual-behavioral perspective. This approach offers a scientific foundation for the detailed design of child-friendly streets.

In terms of research methods, existing literature primarily employs qualitative, quantitative, or mixed-methods approaches to assess the child-friendliness of urban streets. Quantitative research often relies on the construction of structured indicator systems and weighting analyses, covering dimensions such as accessibility, safety, comfort, and activity levels, and frequently uses methods like AHP, TOPSIS, and fuzzy comprehensive evaluation to score different street samples [7]. Qualitative research, on the other hand, emphasizes behavioral observations and subjective feedback, often collecting perceptual data from children and their caregivers through questionnaires, interviews, mapping, or participatory design to reflect the non-material experiences of street spaces [6]. In recent years, mixed methods have become a growing trend, with researchers attempting to integrate streetscape imagery, GIS data, and behavioral data to enhance the objectivity and multidimensional recognition capabilities of spatial analysis [5]. Concurrently, some studies have introduced technological tools, such as visual environment recognition based on multi-source streetscape images, spatial preference analysis using eye-tracking, and behavioral trajectory collection via mobile devices, driving child-friendly street research toward data-driven and intelligent approaches [19]. However, overall, the field still predominantly relies on small-scale, localized, and low-frequency manual surveys, lacking an automated evaluation framework for citywide and large-scale street systems. This limitation makes it difficult to meet the demands of dynamic governance in the context of rapid urban renewal.

### 2.2 Deep learning in urban street studies

In recent years, with the rapid accumulation of street-view imagery data, deep learning has emerged as a pivotal methodological tool for urban street studies. Unlike traditional approaches that rely on questionnaires, manual scoring, and on-site observations, deep learning can automatically extract complex semantic information from imagery and spatial data, thereby enabling efficient recognition and quantification of street environmental quality, visual characteristics, and human perception [20]. The adoption of this technology has facilitated a paradigm shift from subjective experience-based evaluation toward data-driven intelligent perception, offering new avenues for the integrated study of street environments, behavioral patterns, and socio-psychological mechanisms [21].

In the field of street-view visual perception, convolutional neural networks (CNNs) have become the most widely applied deep learning architecture [22]. Through semantic segmentation, feature extraction, and classification of street-view images, researchers have achieved automated recognition of multidimensional features such as street greenness, visual openness, façade color composition, sky visibility, and traffic elements [23]. Kruse et al. [8] quantified the urban playability index from Google Street View imagery using deep learning models, revealing the latent relationship between physical street attributes and children’s behavioral activities. Zhang and Huang et al. [19] employed a ResNet-based framework to analyze the color characteristics of urban streets and developed a predictive model of psychological comfort in street spaces. Wu et al. [9] further integrated semantic segmentation with affective recognition techniques to quantify the restorative features of streetscapes, offering new theoretical insights into the perceptual differences between children and adults regarding urban visual environments. Collectively, these studies demonstrate that deep learning–based street-view analysis can capture perceptually salient factors from the visual dimension, providing an objective data foundation for assessing street-level friendliness.

Beyond visual feature recognition, recent studies have begun to integrate deep learning with multi-source street-level data to establish comprehensive evaluation frameworks. Graph neural networks (GNNs) and multimodal neural networks (MNNs) have been employed to fuse street-view imagery, geographic information system (GIS) data, sensor data, and traffic flow information, thereby uncovering the intricate relationships among spatial structure, human perception, experience, and behavior [24,25]. Tang et al. [26] constructed a city-scale walkability model based on the fusion of street-view imagery and road-network data, achieving fine-grained identification of street-level spatial functions. Yao et al. [27] proposed a human–machine adversarial scoring framework that combines deep learning with expert feedback for the evaluation of urban safety perception and livability. Yang et al. [28]integrated the empathy-based stories method with deep learning to assess the child-friendliness of streets in Nanjing, identifying key visual elements influencing children’s perceptions. Although these studies advance methodological diversity in street-environment analysis, most existing models remain confined to static image–based feature recognition and lack a direct mapping to residents’ subjective perceptions.

Overall, deep learning has achieved substantial progress in urban street research. On one hand, the introduction of CNNs, GNNs, and related architectures has significantly improved the automation and precision of street-view analysis, promoting a shift from qualitative, human-dependent evaluations toward quantitative semantic interpretation of urban spaces. On the other hand, such models facilitate the integration of heterogeneous multi-source data, supporting a holistic representation of street environments that bridges physical attributes and perceptual dimensions [29]. Nevertheless, existing research predominantly focuses on the extraction of objective visual features, while the abstract dimensions of streets, such as architectural style, color ambiance, and emotional perception, remain underexplored. Moreover, most perceptual evaluation frameworks are constructed from an adult-centered perspective, with limited attention to the perceptual mechanisms and experiential patterns specific to children.

## 3. Methods

### 3.1 Framework design of the evaluation system

This study takes urban streets in Shanghai as an example and aims to propose a general method for constructing a child-friendliness assessment system for urban streets. This method is based on deep learning technology and integrates two key types of data: one is comprehensive urban street feature data, and the other is children’s perception evaluation data of the corresponding urban streets’ child-friendliness. As mentioned earlier, the complete urban street features include concrete and abstract features. Concrete feature data can be directly represented numerically, while abstract feature data cannot be directly quantified and are represented by one or more street-view photographs. Additionally, due to varying lengths of urban streets, the number of street-view photographs used to represent the abstract features of each urban street sample may differ.

**Fig 1:**
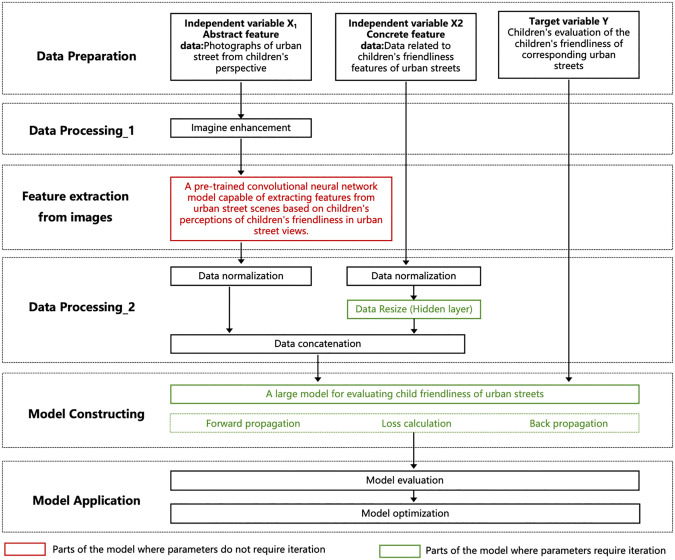
Framework of the Urban Street Child-Friendliness Assessment System. Image source: self-drawn by the author.

To address the characteristics of the aforementioned data, this study proposes the following model scheme: First, a convolutional neural network model is pre-trained to extract corresponding urban street scene features based on children’s evaluations of the child-friendliness of urban street scenes. Then, each urban street scene image in the urban street samples is input into the trained convolutional neural network for child-friendliness prediction, and the output of the adaptive average pooling layer is used as the feature data of that street scene. If the abstract features of an urban street sample consist of multiple street view photos, the feature data of each street scene in the sample is extracted sequentially using the above method, and the corresponding feature vectors of all street scene data are averaged to achieve feature fusion of multiple street view photos, thereby obtaining the final abstract feature data of the urban street sample. This method ensures that regardless of the number of street view photos, the final street scene feature data for each urban street sample has the same dimensions, facilitating subsequent training. In this study, the number of abstract features extracted through the pre-trained convolutional neural network exceeds the number of manually collected concrete features. If the two are directly concatenated for training, the model’s predictions would be more influenced by the abstract features of urban streets. To ensure a balanced extraction of both types of features, this study first processes the concrete feature data of urban streets through a hidden layer to expand its dimensions to match those of the extracted abstract feature data. The expanded concrete feature data is then concatenated with the extracted abstract feature data, and the concatenated data is finally input into the subsequent neural network for training (Fig 1).

Based on the above scheme, this study constructs two models. One is a pre-trained convolutional neural network model used to extract urban street scene features. For the training of this model, a dataset for the pre-trained convolutional neural network model is collected and compiled. The other is the final urban street child-friendliness evaluation model constructed in this study, used to assess the child-friendliness of urban streets. For the training of this model, a dataset for the urban street child-friendliness evaluation model is collected and compiled.

### 3.2 Child-friendly urban street model dataset – sample selection of urban streets

In this study, urban streets are delineated by intersections, with the street segment between two intersections considered as one urban street. Considering that even for the same urban street, the street elements on either side may differ, and such differences in elements may lead to variations in children’s evaluations, this study separately collects concrete and abstract data for both sides of each urban street and independently records their child-friendliness evaluation results to ensure the authenticity and reliability of the research data. Ultimately, each urban street generates two street sample datasets for use in model training in this study.

**Fig 2:**
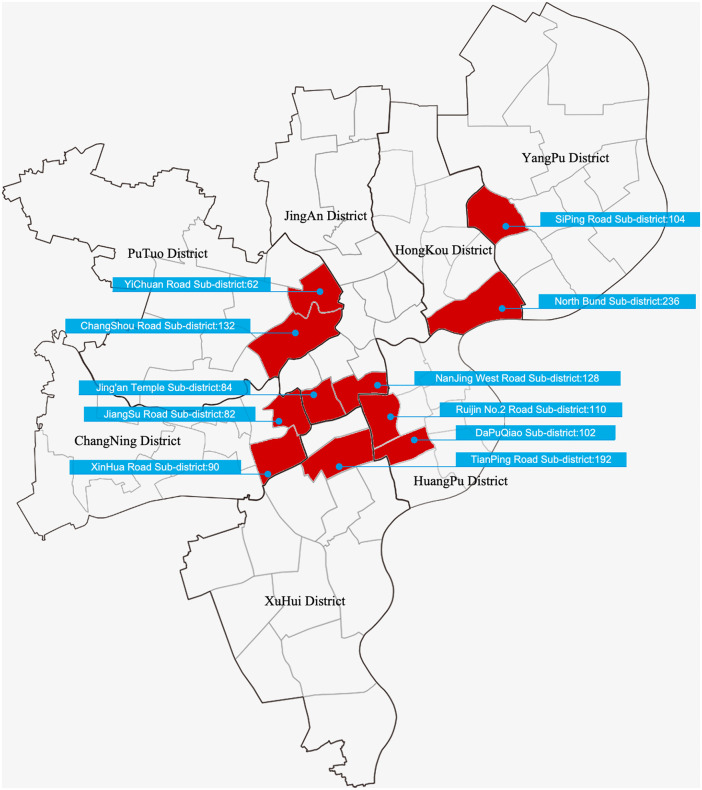
Schematic distribution map of urban street research samples in Shanghai urban area. Image source: self-drawn by the author. This diagram is for illustrative purposes only and may differ from the original image.

Additionally, all urban street sample data in this study are sourced from the urban area of Shanghai. As one of China’s most representative cities, Shanghai ensures the consistency of the study and the comparability of the data [30]. To maximize coverage of diverse community types and child groups across different socioeconomic backgrounds, sampling locations are determined by fully accounting for the distribution of various community types and population composition characteristics in Shanghai. A total of 1,322 urban street samples are selected, with the specific distribution as follows: 82 from Jiangsu Road Subdistrict, Changning District; 90 from Xinhua Road Subdistrict, Changning District; 236 from North Bund Subdistrict, Hongkou District; 102 from Dapuqiao Subdistrict, Huangpu District; 110 from Ruijin No.2 Road Subdistrict, Huangpu District; 84 from Jing’an Temple Subdistrict, Jing’an District; 128 from West Nanjing Road Subdistrict, Jing’an District; 62 from Yichuan Road Subdistrict, Putuo District; 132 from Changshou Road Subdistrict, Putuo District; 192 from Tianping Road Subdistrict, Xuhui District; and 104 from Siping Road Subdistrict, Yangpu District (Fig 2).

These street samples encompass not only diverse community types but also a heterogeneous social structure ranging from high-income to low-income households and from local-resident-dominated to migrant-population-mixed neighborhoods, characterized by three typical categories:

First, Jiangsu Road and Xinhua Road Subdistricts in Changning District, Dapuqiao and Ruijin No.2 Road Subdistricts in Huangpu District, and Tianping Road Subdistrict in Xuhui District. These areas consist primarily of a large number of old lanes, interspersed with a small number of high-rise commercial buildings. The lane spaces are typically inhabited by a large aging population and original residents, while also attracting some migrant worker families due to relatively low rental costs, reflecting a mixed-class residential pattern.

Second, North Bund Subdistrict in Hongkou District, and Jing’an Temple and West Nanjing Road Subdistricts in Jing’an District. These areas are lined with high-rise buildings, featuring modern commercial office buildings and upscale residences. They mainly represent the living environments of middle-to-high-income families and “middle-class” children, with an overall modern streetscape and well-maintained facilities.

Third, Yichuan Road and Changshou Road Subdistricts in Putuo District, and Siping Road Subdistrict in Yangpu District. These areas evolved from “worker’s new villages” constructed between the 1950s and 1980s, exhibiting distinct characteristics of being “old, small-scale, and dilapidated”. According to Shanghai’s census data and community characteristic analysis, these areas are currently typical settlements for middle-to-low-income groups and migrant workers, characterized by high population density and mobility. Including these areas in the sample effectively captures the authentic perceptions of children from non-privileged backgrounds toward street spaces.

Through this geospatial stratified sampling strategy, the study avoids survivorship bias to a certain extent, ensuring that the assessment model reflects not only the preferences of children from elite communities but also the perspectives of those from marginalized and low-income neighborhoods.Regarding the studied child group, it also should be noted that although different countries have varying standards for defining childhood age groups, literature reviews and field surveys indicate that children aged 7–12 exhibit a pronounced need for independent participation in street life in daily routines. They also possess basic logical thinking skills, can understand and complete questionnaires, and demonstrate a certain level of cognitive autonomy [31]. Therefore, this study primarily targets children in this age group.

### 3.3 Child-friendly urban street model dataset – concrete feature data of urban street spaces

Urban street spaces are typically composed of elements such as sidewalks, non-motorized vehicle lanes, motorized vehicle lanes, street-side buildings, landscape greenery, activity areas, and service facilities. In this study, 10 experts in child-friendly urban design were invited to analyze child-friendly features of urban streets. Combining their insights with findings from prior research and on-site investigations, a total of 50 concrete child-friendly street feature data points were summarized, all of which can be quantified statistically. Since deep learning models can automatically extract features from raw data without requiring additional manual feature selection, all these urban street features were retained and categorized according to street elements to form the concrete features related to child-friendliness (Table 1). This urban street information constitutes the concrete feature data in the dataset for the child-friendliness evaluation model of urban streets.

**Table 1:** Statistical table of child-friendly concrete features in urban streets.

Urban Street Element	Child-Friendliness Related Feature	Feature Quantification Method
Sidewalk	Sidewalk Width	Average effective pedestrian passage width (unit: meters)
	Sidewalk Length	Straight-line distance between intersections (unit: meters)
	Degree of Sidewalk Encroachment	The ratio of encroached sidewalk length to total sidewalk length (0% = 0, 0–20% = 1, 20–40% = 2, 40–60% = 3, 60–80% = 4, 80–100% = 5)
	Sidewalk Damage Level	The ratio of damaged sidewalk length to total sidewalk length (0% = 0, 0–20% = 1, 20–40% = 2, 40–60% = 3, 60–80% = 4, 80–100% = 5)
	Sidewalk Cleanliness Level	Ratio of smooth, clean sidewalk length free from illegal parking (0% = 0, 0–20% = 1, 20–40% = 2, 40–60% = 3, 60–80% = 4, 80–100% = 5)
	Proportion of Barriers Isolating Sidewalk from Other Lanes	(0% = 0, 0–20% = 1, 20–40% = 2, 40–60% = 3, 60–80% = 4, 80–100% = 5)
Bicycle Lane	Presence of Bicycle Lane	Present = 1/ Absent = 0
	Bicycle Lane Width	Average effective passage width (unit: meters)
	Bicycle Traffic Volume	Peak-period bicycle flow rate (units: vehicles per minute)
Motor Vehicle Lane	Motor Vehicle Lane Width	Average effective passage width (unit: meters)
	Number of Motor Vehicle Lanes	Count (units: number)
	Motor Vehicle Traffic Condition	Smooth = 1, Slow-moving = 2, Congested = 3, Severe Congestion = 4
	Average Vehicle Speed	0–40 km/h = 1, 40–60 km/h = 2, 60–80 km/h = 3, > 80 km/h = 4
	Presence of Safety Islands	Present = 1/ Absent = 0
	Presence of Pedestrian Warning Systems	Present = 1/ Absent = 0
	Presence of Pedestrian Overpasses	Present = 1/ Absent = 0
	Presence of Elevated Road Above	Present = 1/ Absent = 0
	Traffic Lights at Intersections	One intersection with lights = 2/ Both intersections = 1/ None = 0
Street-Front Buildings	Average Building Height	Average height (unit: meters)
	Number of Motor Vehicle Entrances/Exits	Count (units: number)
	Proportion of Arcade/Overhanging Space	(0% = 0, 0–20% = 1, 20–40% = 2, 40–60% = 3, 60–80% = 4, 80–100% = 5)
	Presence of Objects Intruding into Street	Present = 1/ Absent = 0 (e.g., ground-floor encroachment or risk of falling objects from upper floors)
	Proportion of Commercial Buildings	0% = 0, 0-20% = 1, 20-40% = 2, 40-60% = 3, 60-80% = 4, 80-100% = 5
	Proportion of Residential Buildings	0% = 0, 0-20% = 1, 20-40% = 2, 40-60% = 3, 60-80% = 4, 80-100% = 5
	Proportion of Cultural and Educational Buildings	0% = 0, 0-20% = 1, 20-40% = 2, 40-60% = 3, 60-80% = 4, 80-100% = 5
	Proportion of Sports Facilities Buildings	0% = 0, 0-20% = 1, 20-40% = 2, 40-60% = 3, 60-80% = 4, 80-100% = 5
	Proportion of Medical Facilities Buildings	0% = 0, 0-20% = 1, 20-40% = 2, 40-60% = 3, 60-80% = 4, 80-100% = 5
	Proportion of Office Buildings	0% = 0, 0-20% = 1, 20-40% = 2, 40-60% = 3, 60-80% = 4, 80-100% = 5
Landscape and Greenery	Proportion of Street Trees	0% = 0, 0-20% = 1, 20-40% = 2, 40-60% = 3, 60-80% = 4, 80-100% = 5
	Proportion of Child-Interactive Landscapes	0% = 0, 0-20% = 1, 20-40% = 2, 40-60% = 3, 60-80% = 4, 80-100% = 5
	Proportion of Other Landscape Features	0% = 0, 0-20% = 1, 20-40% = 2, 40-60% = 3, 60-80% = 4, 80-100% = 5
Activity Spaces	Proportion of Usable Activity Space	0% = 0, 0-20% = 1, 20-40% = 2, 40-60% = 3, 60-80% = 4, 80-100% = 5
	Area of Usable Activity Space	0 m² = 0, 0–20 m² = 1, 20–40 m² = 2, 40–60 m² = 3, 60–80 m² = 4, > 80 m² = 5
Service Facilities	Proportion of Bicycle Parking Spaces	0% = 0, 0-20% = 1, 20-40% = 2, 40-60% = 3, 60-80% = 4, 80-100% = 5
	Proportion of Motor Vehicle Parking Spaces	0% = 0, 0-20% = 1, 20-40% = 2, 40-60% = 3, 60-80% = 4, 80-100% = 5
	Lighting System Coverage Rate	0% = 0, 0-20% = 1, 20-40% = 2, 40-60% = 3, 60-80% = 4, 80-100% = 5
	Surveillance System Coverage Rate	0% = 0, 0-20% = 1, 20-40% = 2, 40-60% = 3, 60-80% = 4, 80-100% = 5
	Proportion of Barrier-Free Facilities	0% = 0, 0-20% = 1, 20-40% = 2, 40-60% = 3, 60-80% = 4, 80-100% = 5
	Proportion of Fitness Trails	0% = 0, 0-20% = 1, 20-40% = 2, 40-60% = 3, 60-80% = 4, 80-100% = 5
	Number of Fitness Facilities	Count (units: number)
	Number of Play Facilities	Count (units: number)
	Number of Rest Facilities	Count (units: number)
	Number of Road Signs	Count (units: number)
	Number of Transit Stops	Count (units: number)
	Number of Drinking Fountains	Count (units: number)
	Number of Public Toilets	Count (units: number)
Other Elements	Proportion of Non-Motorized Vehicle Parking on Street	0% = 0, 0-20% = 1, 20-40% = 2, 40-60% = 3, 60-80% = 4, 80-100% = 5
	Proportion of Motor Vehicle Parking on Street	0% = 0, 0-20% = 1, 20-40% = 2, 40-60% = 3, 60-80% = 4, 80-100% = 5
	Presence of Odor on Street	Present = 1/ Absent = 0
	Street Sound Environment	Noisy(>60dB) = 2/ Normal (40-60dB) = 1/ Quiet (<40dB) = 0

### 3.4 Child-friendly urban street model dataset – abstract feature data of urban street spaces

In this study, the abstract features of urban streets are recorded using street-view images. Since sidewalks are the primary spaces where children engage in street life and are located in the core area of all street elements, photographers are required to stand in the middle of the sidewalk on one side of the urban street and capture street-view photos facing forward. This ensures a complete representation of the cross-sectional street-view information for a segment of the urban street sample.

**Fig 3:**
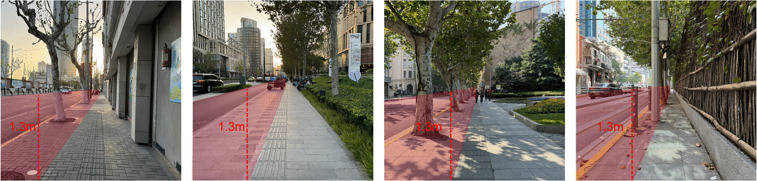
Example of street view photos of urban street taken on site. Image source: Taken by the author on site.

Additionally, as the core focus of this research is the perception of urban street friendliness from a child’s perspective, the shooting height of the street-view photos must closely align with the actual viewpoint of children. As mentioned earlier, the research subjects of this paper are mainly children aged 7–12 years. According to the 2024 Report on the Height Status of Chinese Children published by the China Children and Teenagers’ Fund [32], the average height of Chinese children aged 7–12 is approximately 1.35 meters. Considering that children’s eye level, when looking straight ahead, is slightly lower than their height, this study sets the shooting height of street view images at approximately 1.3 meters (Fig 3) to best simulate their actual visual experience on the streets.

Typically, the abstract features of one side of an urban street require multiple street view images to represent. During the research process, the optimal range for capturing street view images was determined through field surveys. Specifically, each photo should clearly cover the street environment within a 50-meter radius from the shooting point, as areas beyond this distance suffer from reduced resolution, compromising assessment accuracy. Therefore, in this study, street view images were captured at 50-meter intervals starting from the beginning of one side of each street. However, since some urban streets exhibit highly similar visual features, street view photos with excessive similarity were filtered out when finalizing the selection for each urban street sample. Ultimately, from the collected data of 1,322 urban street samples, a total of 6,724 street view photos were selected. These urban streetscapes constitute the abstract feature data for the urban street samples in the dataset of the child-friendliness evaluation model.

### 3.5 Child-friendly urban street model dataset – child-friendliness evaluation indicator design

To quantify children’s perception of urban street friendliness, this study employs a five-point Likert scale for scoring child-friendliness indicators. Here, 1 point represents the worst experience for the indicator, 2 points for poor, 3 for average, 4 for good, and 5 for excellent. During the entire survey period, 14 researchers were assigned to conduct questionnaire surveys among children aged 7–12 in urban streets of Shanghai. The survey was carried out from September 24, 2024 to March 31, 2025, primarily during school dismissal hours on weekdays and daytime periods on weekends. In total, this study effectively collected child-friendliness scores for 1,322 urban streets in Shanghai. Informed consent was obtained from all subjects involved in the study. The minors who filled out the questionnaire obtained consent from their parents or guardians.Each survey questionnaire includes a consent statement. The first question in the questionnaire survey of this study was: “Do you consent to your or your child’s participation in this research?” So, all such consents were obtained in written form.Additionally,the study was conducted in accordance with the Declaration of Helsinki, and approved by the Institutional Review Board of the College of Architecture and Urban Planning, Tongji University, authorization number: 2024092301.

For each urban street sample, this study randomly selected 20 children aged 7–12 who were active in the area to participate in a questionnaire survey. Due to the potential for significant individual differences in children’s subjective perceptions, and to ensure the objectivity of the evaluation and the reliability of the data, the 20 ratings collected for each urban street sample must meet the following requirements. First, the number of identical ratings among the 20 ratings for the entire street is counted, requiring that the number of identical ratings be greater than or equal to 12. This ensures that over 60% of the children have consistent evaluation results for the street. This requirement is primarily based on field research findings, which indicate that setting the threshold for identical ratings at 12 can ensure both the uniformity of sample ratings and an adequate sample size. If the threshold were raised, the number of eligible samples would significantly decrease. Second, the overall standard deviation of the 20 ratings is calculated, requiring it to be less than or equal to 0.7. This ensures that in extreme cases where the number of identical ratings is only 12, the difference between the other ratings and the most frequent rating does not exceed 1. It also guarantees that in datasets where the number of identical ratings is 12 or more, the rounded mean of all ratings matches the value of the most frequent rating. These two steps ensure that the dispersion of the rating dataset is not excessively large, thereby making the evaluation results valid and usable. If the rating dataset for a particular street does not meet these two requirements, the evaluation data for that street will be excluded or re-surveyed offline. Ultimately, the most frequent rating value in the dataset is taken as the final child-friendliness rating for the street.

After dual verification, the final number of urban street samples with different child-friendliness ratings that can be used for training the urban street child-friendliness assessment model is shown in the table below (Table 2). Each urban street sample includes concrete feature data, abstract feature data, and the corresponding child-friendliness rating.

**Table 2:** Statistical table of the number of urban street samples with different child-friendliness ratings in the urban street child-friendliness assessment model dataset (before data augmentation).

Child-friendliness score	1 point	2 points	3 points	4 points	5 points	SUM
SUM	157	294	488	268	115	1322

### 3.6 Pre-trained convolutional neural network model dataset

In the model architecture designed for this study, it is necessary to pre-train a convolutional neural network model capable of extracting corresponding urban street view features based on children’s child-friendliness evaluations of urban streets. This model dataset requires two types of data: one is urban street view photo data, and the other is children’s child-friendliness ratings for these urban street views.

Regarding the urban street view photo data, since this convolutional network is used to extract abstract features of urban street samples, the street view images used to train this convolutional network must also meet the requirements outlined in Section 3.3. To save research time and costs, this study directly selected the required urban street view photos from the urban street view dataset constructed in Section 3.3 (Fig 2).

As for the children’s child-friendliness ratings for each selected urban street view, since each urban street view can only represent a certain segment of the corresponding street and not the entire street, the child-friendliness ratings for the entire street collected in Section 3.4 cannot be applied to these urban street view images. The child-friendliness ratings for the street segments corresponding to these street views need to be re-surveyed. For each urban street segment corresponding to a street view image, this study randomly selected 20 children aged 7–12 who were active in the area to participate in a questionnaire survey to obtain their child-friendliness ratings for that urban street segment. Although these ratings differ from the child-friendliness ratings for the entire urban street, the 20 ratings collected for each urban street segment must also meet the two requirements specified in Section 3.4.

The data collection for this dataset was conducted simultaneously with the data collection for the urban street child-friendliness evaluation model dataset mentioned earlier. A total of 14 researchers were assigned to conduct questionnaire surveys among children aged 7–12 in urban streets of Shanghai. The survey was carried out from September 24, 2024 to March 31, 2025, primarily during school dismissal hours on weekdays and daytime periods on weekends. In total, this study effectively collected child-friendliness ratings for 1,178 urban street segments corresponding to urban street views in Shanghai (Table 3).

**Table 3:** Statistical table of the number of urban street-view samples with different child-friendliness ratings in the pre-trained CNN model dataset (before data augmentation).

Child-friendliness score	1 point	2 points	3 points	4 points	5 points	SUM
SUM	148	226	431	258	115	1178

## 4. Deep learning-based automatic assessment model

### 4.1 Preprocessing of the pre-trained convolutional neural network model dataset.

For the pre-trained convolutional neural network model, the distribution of street-view images across different rating levels in the dataset exhibits significant imbalance (Table 3). This class imbalance issue may lead the deep learning model to predominantly learn features from categories with larger sample sizes during training, while showing weaker recognition capability for categories with fewer samples. Additionally, such imbalance could make the model more sensitive to input variations, thereby reducing its generalization ability and robustness.

**Fig 4:**
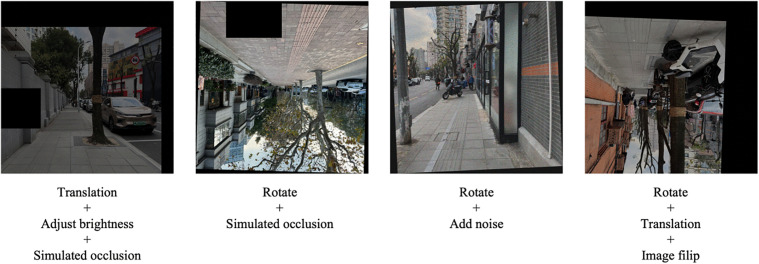
Urban streetscape photos after image augmentation in the pre-trained convolutional neural network model dataset. Image source: Image transformation performed by computer.

To ensure balanced learning capability across different child perception rating categories, this study employs image augmentation techniques to optimize the street-view image dataset. Image augmentation generates new samples by applying a series of transformations to original images, including Rotation, Translation, Mirror flipping, Brightness adjustment, Noise addition Simulated occlusion (Fig 4). These augmentation operations not only balance the sample quantities across rating categories but also enhance dataset diversity. This enables the model to better adapt to street-view images under various environmental conditions, improving both its generalization capability and robustness when handling complex scenarios.

After image enhancement processing, the dataset for this convolutional neural network model was expanded to 4,233 streetscape images (Table 4), constituting a medium-sized image dataset. To ensure proper data partitioning and optimize model performance, this study adopted a three-stage division strategy comprising training, validation, and test sets. The training set was used to ensure the model could thoroughly learn the patterns and features within the data. The validation set was employed during the training process to evaluate model performance, fine-tune hyperparameters, and prevent overfitting. The test set was reserved for the final assessment of the model’s generalization capability, ensuring robust predictive performance on unseen data.

**Table 4:** Statistical table of the number of urban streetscape samples with different child-friendliness ratings in the pre-trained convolutional neural network model dataset (after data augmentation).

Child-friendliness score	1 point	2 points	3 points	4 points	5 points	SUM
SUM	888	904	862	774	805	4233

### 4.2 Preprocessing of the urban street child-friendliness evaluation model dataset

**Fig 5:**
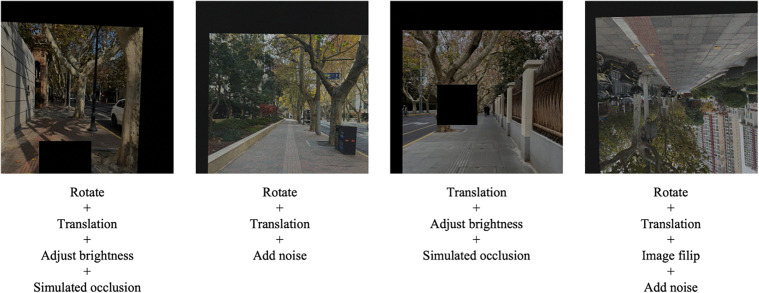
Urban streetscape photos after image augmentation in the urban street child-friendliness assessment model dataset. Image source: Image transformation performed by computer.

For the urban street child-friendliness assessment model ultimately constructed in this study, the distribution of urban street samples across different rating levels in the dataset is also imbalanced (Table 2). Therefore, this study similarly requires dataset expansion. Since the abstract feature data in this dataset is more amenable to data augmentation operations compared to the concrete feature data, this study selectively employs image augmentation methods to apply a series of transformations to the street view photos in the urban street samples. These transformations include rotation, translation, mirror flipping, brightness adjustment, noise addition, and simulated occlusion, among others (Fig 5). These augmented images are then processed through the pre-trained convolutional neural network model to extract the abstract feature data of the urban streets. Although the concrete feature data of the urban streets remains unchanged, the final concatenated complete street data still differs, thereby achieving dataset expansion.

After data augmentation processing, the dataset for the urban street child-friendliness assessment model was expanded to 4,524 urban street samples (Table 5). This dataset was similarly divided into training set, validation set, and test set.

**Table 5:** Statistical table of urban street samples with different child-friendliness rating levels in the urban street child-friendliness assessment model dataset (after data augmentation).

Child-friendliness score	1 point	2 points	3 points	4 points	5 points	SUM
SUM	942	882	976	804	920	4524

Additionally, the abstract feature data extracted by the pre-trained convolutional neural network model and the manually collected concrete feature data have inconsistent units of measurement. Even within the concrete feature data, the units of measurement for individual feature values are not entirely uniform. This results in significant numerical disparities between some features. Feeding such data directly into a deep learning model can make the model difficult to train, as features with larger numerical ranges may dominate distance calculations, thereby diminishing the importance of other features. To address this, the data must undergo dimensionless normalization. Given that the numerical differences within each feature in this study are relatively small, with no significant outliers and most data ranges being fixed, the Min-Max normalization method (Min-Max Normalization) was chosen to standardize the data for each feature, transforming it into a distribution within the range [0, 1]. The maximum and minimum values for this Min-Max normalization were selected from the complete dataset and uniformly applied to the training, validation, and test sets.

### 4.3 Design and evaluation of pre-trained convolutional neural network model

In the architectural design of this study’s model, this study first utilizes street view images from the pre-trained CNN model dataset along with their corresponding child-friendliness ratings to pre-train a convolutional neural network model. This model is designed to extract urban streetscape features based on children’s evaluations of streetscape child-friendliness. Considering the relatively small scale of image samples for CNN training and the moderate computational requirements for the five-category classification task, this study selected six mainstream CNN architectures with varying depths - VGG16, Inception_v1, Inception_v3, ResNet18, ResNet34, and ResNet50 – to prevent overfitting caused by excessive network parameters. A comparative analysis of these models’ training performance was conducted to determine the most suitable architecture for our research task.

This study first divided the dataset of 4,233 streetscape images into an 80% temporary dataset and a 20% test set. Then, the Stratified K-Fold algorithm (with the n_splits parameter set to 5) was applied to the temporary dataset to further split it into an 80% training set and a 20% validation set, thereby completing cross-validation for the model. During each dataset split, care was taken to ensure that the distribution proportions of different categories remained consistent across all datasets.

To ensure training stability, the hyperparameters of all models were kept consistent. The batch size for training data was set to 32, the Adam optimizer was employed with a learning rate of 0.001, and the cross-entropy loss function (Cross-Entropy Loss) was used for optimization. The total number of training epochs was set to 100. After each epoch, the model parameters were saved, and performance was evaluated on the validation set to monitor the model’s convergence and generalization ability.

**Fig 6:**
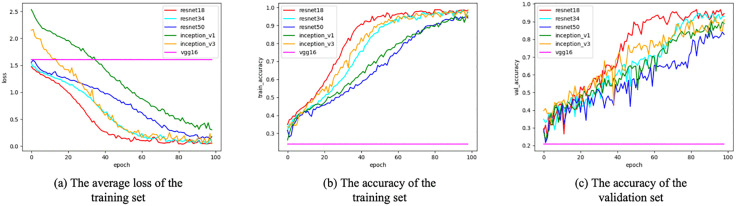
Experimental data plot of pre-trained convolutional neural network models on training and validation sets. Image source: self-drawn by the author.

The experimental results of the model during the training and validation phases are shown in Fig 6. On the training set, the ResNet18, ResNet34, and Inception_v3 models converged quickly, with their loss values stabilizing after 60 epochs and their accuracy approaching optimal levels, while other models exhibited overfitting or slow convergence. On the validation set, ResNet18, ResNet34, and Inception_v3 continued to perform exceptionally well, demonstrating not only strong classification capabilities but also robust generalization abilities. Among them, the ResNet18 network performed the best. After five rounds of cross-validation training, the model achieved the lowest average loss value of 0.0403, while the highest average accuracy rates on the training and validation sets reached 98.75% and 96.86%, respectively.

During the testing phase, this study selected the model with the best overall performance on the training and validation sets for evaluation. In addition to calculating the overall classification accuracy of the model, this paper also focused on the recall and precision rates for each score category. Recall represents the proportion of images with a specific score that the model correctly classified, while precision indicates the proportion of samples predicted by the model as a certain score that truly belong to that category.

The experimental results are shown in Table 6. The ResNet18 model also performed the best on the test set, maintaining a high accuracy of 96.38%. The model exhibited strong performance in both recall and precision. For street-view data with a score of 1, the recall rate reached 98.64% with 100% precision, while for data with a score of 2, the recall rate was 100% with 96.06% precision. This indicates that the model can accurately identify street scenes with low child-friendly scores while minimizing misclassification. These experimental results fully demonstrate that the ResNet18 network is the most suitable model architecture for extracting abstract urban street features in this study.

**Table 6:** Experimental data table of pre-trained convolutional neural network model (best performing model on validation sets) on test set.

	VGG16	Inception_v1	Inception_v3	ResNet18	ResNet34	ResNet50
Accuracy	19.21%	82.05%	94.49%	96.38%	93.86%	85.67%
Recall (1 Point)	0.00%	90.48%	96.60%	98.64%	97.28%	91.84%
Precision (1Point)	0.00%	91.10%	97.26%	100.00%	96.62%	93.10%
Recall (2 Points)	100.00%	82.79%	95.90%	100.00%	94.26%	85.25%
Precision (2Points)	19.21%	81.45%	92.13%	96.06%	94.26%	84.55%
Recall (3 Points)	0.00%	84.75%	90.68%	93.22%	90.68%	85.59%
Precision (3Points)	0.00%	73.53%	89.92%	97.35%	90.68%	75.94%
Recall (4 Points)	0.00%	66.93%	93.70%	92.91%	92.13%	72.44%
Precision (4Points)	0.00%	76.58%	93.70%	92.19%	92.13%	82.14%
Recall (5 Points)	0.00%	84.30%	95.04%	100.00%	94.21%	92.56%
Precision (5Points)	0.00%	86.44%	99.14%	95.90%	95.00%	91.80%

### 4.4 Design and evaluation of the urban street child-friendliness assessment model

The trained pre-trained convolutional neural network model can be used to extract abstract data features of urban street samples from the child-friendliness assessment dataset, as detailed in Section 3.1. After obtaining the abstract feature data of urban street samples, it can be concatenated with the concrete feature data of the samples and fed into a subsequent artificial neural network for training. Since the number of street-view features extracted by the ResNet18 convolutional neural network is 512, while the number of concrete features of urban streets is only 50, directly concatenating the two would result in the trained model being more influenced by street-view features. To ensure a balanced contribution from both feature types, this study first processes the concrete feature data of urban streets through a hidden layer to expand its dimension to 512. The expanded concrete feature data is then concatenated with the extracted street-view features, and the combined feature data is finally fed into the subsequent artificial neural network for training.

To ensure rapid convergence of the urban street child-friendliness assessment model and high accuracy on the training and validation set, the study iteratively optimized the model’s network architecture and training parameters. For the network architecture, the model consists of four hidden layers. The first hidden layer processes the concrete feature data of urban street samples, followed by a ReLU activation function, with an input dimension of 50 and an output dimension of 512. The output of this layer is then concatenated with the abstract feature data of urban street samples, resulting in a combined dimension of 1024. The second hidden layer processes the concatenated data, followed by a ReLU activation function, with an input dimension of 1024 and an output dimension of 2048. The third hidden layer takes the output of the second hidden layer, also followed by a ReLU activation function, with an input dimension of 2048 and an output dimension of 4096. The fourth hidden layer processes the output of the third hidden layer and produces the final result, with an input dimension of 4096 and an output dimension of 5. Since the cross-entropy loss function (Cross-Entropy Loss) in Python includes a built-in softmax activation function, no additional activation function is applied to the fourth hidden layer. For the initial hyperparameters, the study sets the batch size to 32, the optimizer to Adam, the learning rate to 0.001, the loss function to cross-entropy loss (Cross-Entropy Loss), and the total training epochs to 100.

In this study, the dataset of 4,524 urban street samples was similarly divided into an 80% temporary dataset and a 20% test set. The Stratified K-Fold algorithm (with the n_splits parameter set to 5) was then applied to the temporary dataset to further split it into an 80% training set and a 20% validation set, thereby completing 5-fold cross-validation for the model. During each dataset split, care was taken to ensure that the distribution proportions of different categories remained consistent across all datasets. After each training epoch, the model parameters are saved, and the model’s performance is evaluated on the validation set to monitor convergence and generalization ability. Once training is completed, the model with the best overall performance on the training and validation sets is tested on the test set to assess its generalization capability, ensuring robust predictive performance on unseen data. The final validated model can then be applied to assess the child-friendliness of unknown urban streets S1 Code.

**Fig 7:**
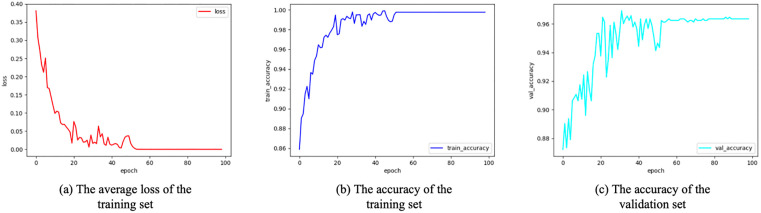
Experimental Data Plot of the urban street child-friendliness assessment model on Training and Validation Sets. Image source: self-drawn by the author.

The experimental results of the model during the training and validation phases are shown in Fig 7. On the training set, both the loss value and accuracy stabilized after 60 epochs, indicating that the model gradually achieved convergence. On the validation set, the overall accuracy continued to show an upward trend, performing comparably to the training set accuracy curve, though with noticeably more fluctuations. After undergoing five rounds of cross-validation training, the evaluation model achieved its lowest average loss value of 0.00399 on the training set, while its highest average accuracy rates reached 99.94% and 96.91% on the training and validation sets, respectively. These results demonstrate that the evaluation model possesses strong learning and generalization capabilities on both the training and validation sets.

During the testing phase, this study selected the model with the best overall performance on the validation sets for evaluation. The experimental results, as shown in Table 7, demonstrate that the model achieved an overall accuracy of 97.35%. Since the primary objective of this study is to accurately identify urban streets with low child-friendliness ratings, particular attention was paid to the classification performance of data rated as 1 and 2 points. According to the experimental results, the model achieved a recall rate of 100% and a precision rate of 99.30% for data rated as 1 point. For data rated as 2 points, the recall rate was 98.48%, and the precision rate was 97.01%. These results indicate that the model has a high probability of correctly identifying low-rated streets while minimizing the misclassification of other-rated streets as low-rated. Therefore, the model can accurately identify urban street spaces with poor child-friendliness, providing a scientific basis for subsequent child-friendly urban improvements.

**Table 7:** Experimental data table of the urban street child-friendliness assessment model (best performing model on validation sets) on test set.

	1 point	2 points	3 points	4 points	5 points
Recall rate	100.00%	98.48%	94.20%	94.89%	99.24%
Accuracy rate	99.30%	97.01%	95.59%	96.30%	98.48%
Overall accuracy rate	97.35%				

### 4.5 Deep learning model analysis

To visualize and quantify the impact of urban street features on the output results of the aforementioned two models, this study employs Grad-CAM technology in the pre-trained convolutional neural network model to visually interpret key elements in the street view data that influence child-friendliness ratings of urban streets. Meanwhile, SHAP technology is applied in the urban street child-friendliness evaluation model to quantitatively explain the key elements in the concrete feature data that affect child-friendliness ratings.

**Fig 8:**
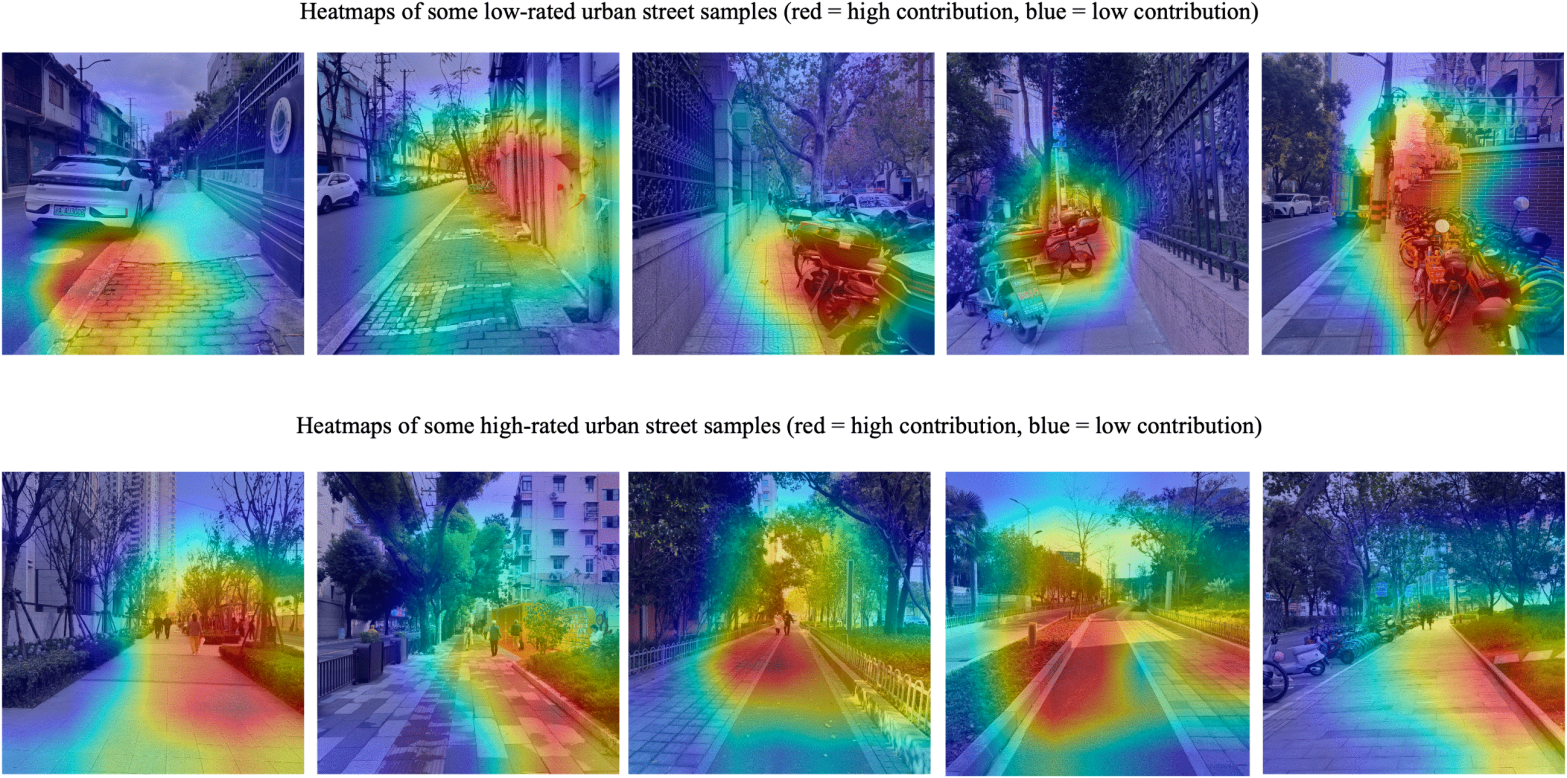
Heat maps of selected urban street scenes in the pre-trained convolutional neural network model. Image source: self-drawn by the author.

For urban street view data representing the abstract features of urban streets, this study adopts the Grad-CAM visualization interpretation technique. By analyzing the “gradient information” of the last convolutional layer in the pre-trained convolutional neural network model (ResNet18 architecture), the image regions that contribute the most to child-friendliness rating decisions are identified and visualized using heatmaps (red areas indicate high-contribution regions, while blue areas represent low-contribution regions). As shown in Fig 8, the elements in urban street scenes that significantly influence child-friendliness rating decisions are primarily concentrated on pedestrian walkways and the spatial interfaces adjacent to them. In low-rated street samples, the typical features captured by the CNN include: damaged sidewalks, lack of clear separation zones, severely obstructed views due to disorderly parked non-motorized vehicles, low greenery coverage, monotonous street colors, simplistic street elements, and the absence of notable gathering nodes. In contrast, high-rated street samples exhibit opposite characteristics, such as clean and well-maintained sidewalks, unobstructed views for children, high greenery coverage, diverse street elements, and the presence of open social nodes.

**Fig 9:**
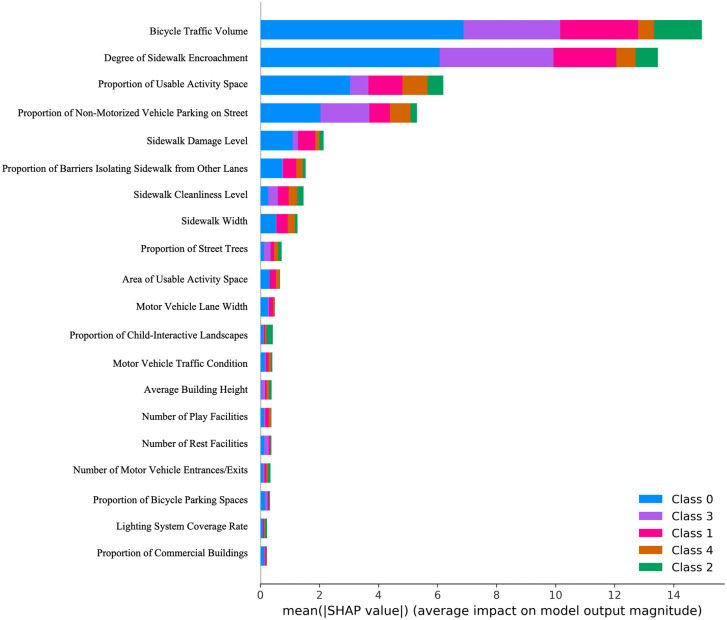
Schematic diagram of the contribution of concrete features in the urban street child-friendliness evaluation model (Top 20 concrete features with the highest contributions). Image source: self-drawn by the author.

For the concrete feature data of urban streets, this study employs the SHAP quantification technique to measure the contribution of each concrete feature to the output of the urban street child-friendliness evaluation model. As shown in Fig 9, the top three concrete features with the greatest impact on the model’s output are non-motorized vehicle traffic flow, the extent of pedestrian path encroachment, and the proportion of activity-friendly spaces. Among these, non-motorized vehicle traffic flow has the most significant impact. Field investigations in this study revealed that a majority of urban streets in Shanghai experience heavy non-motorized vehicle traffic. Some streets lack dedicated non-motorized vehicle lanes, forcing them to share space with pedestrian pathways, which increases safety risks for children. Even on streets with designated non-motorized vehicle lanes, factors such as narrow sidewalks, lack of separation facilities, and riders’ preference for convenience often lead to non-motorized vehicles using pedestrian paths, further endangering children’s safety.Regarding the extent of pedestrian path encroachment, field observations indicate that urban streets in Shanghai frequently face encroachment by non-motorized vehicles, motor vehicles, utility poles, and other obstacles. These encroachments hinder children’s movement and, due to children’s shorter stature, obstruct their sightlines, exacerbating safety hazards.As for the proportion of activity-friendly spaces, the study found that the distance between children’s schools and homes in urban Shanghai is generally short. As a result, children often walk home after school with guardians or peers and are more inclined to linger and play on streets with accessible activity spaces. Consequently, such streets receive higher child-friendliness ratings.

## 5. Verification of Model Generalization Ability Based on Transfer Learning

### 5.1 Sample selection for the transfer learning model

**Fig 10:**
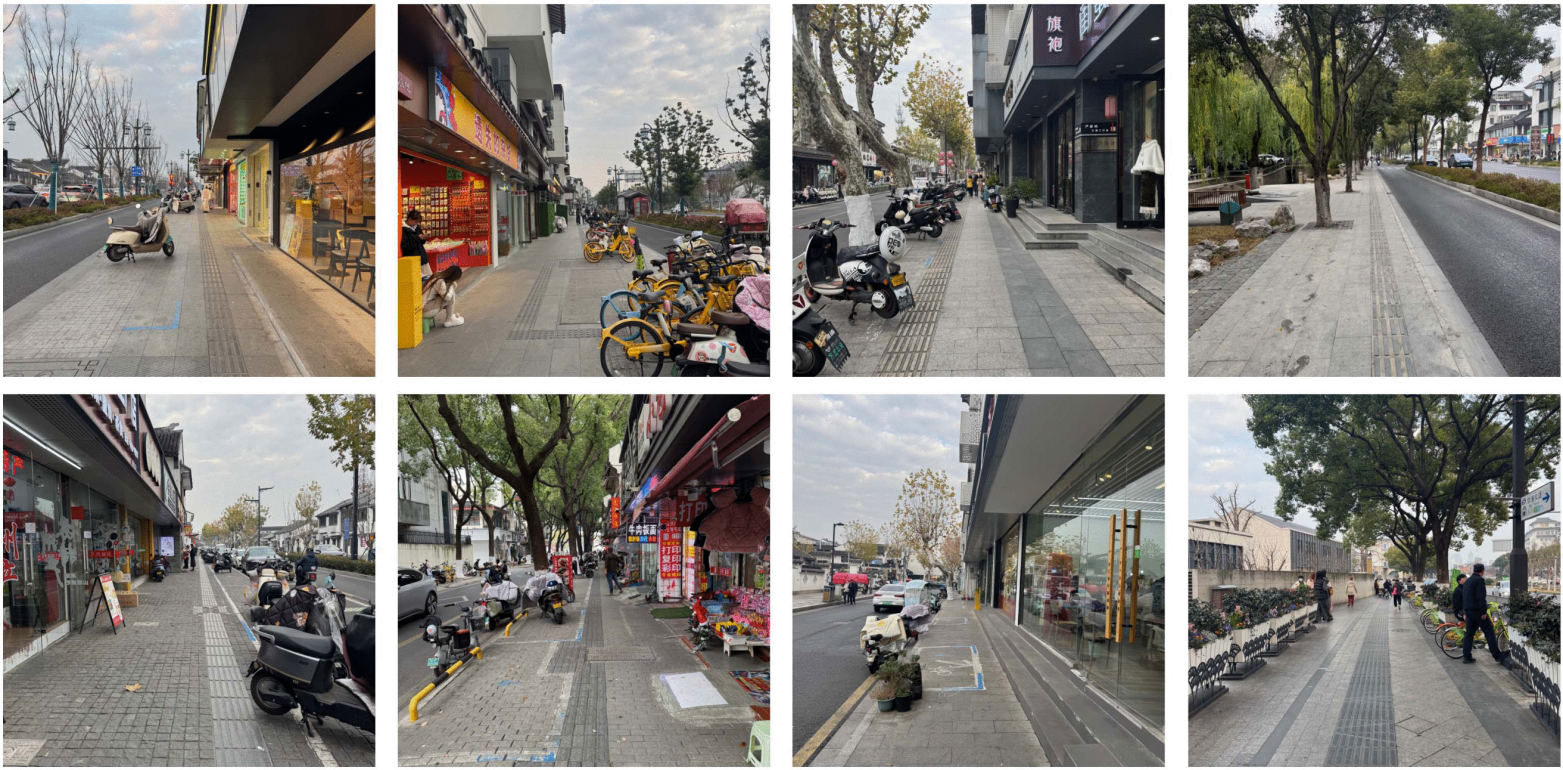
Schematic diagram of urban streets in the ancient city area of Suzhou. Image source: Taken by the author on site.

Despite the sample limitation of using only data from the urban area of Shanghai, partial parameters of the model developed in this study can be transferred to train samples of urban streets with different cultural backgrounds or perceptual assessment samples of diverse child groups via transfer learning, thus meeting the perceptual needs of different child groups in varying urban street contexts. To further verify the generalization ability of the constructed automatic evaluation model across different built environments and child samples, the ancient city area in Gusu District, Suzhou, was selected as the cross-context validation target. Located within Suzhou’s moat, this historic district—hailed as a renowned historical and cultural city—features a mix of traditional Su-style dwellings, Su-style gardens, and modern neo-Su-style architecture (Fig 10). It exhibits significant differences from the urban area of Shanghai in terms of urban form, social composition, and children’s activity scenarios: narrower street scales, more enclosed interfaces, a maximum building height of 24 meters, weaker motor vehicle organization, a predominantly local child population with a lower proportion of migrant workers than Shanghai, among others. These distinguishing features provide an effective test for the model’s applicability under varying conditions of urban form, socioeconomic status, and population demographics.

In the survey of urban streets in the ancient city area of Gusu District, Suzhou, this study collected street view samples for the pre-trained convolutional neural network model dataset and urban street samples for the child-friendliness evaluation model dataset separately, using the identical data collection method applied in the urban area of Shanghai. Considering that transfer learning training does not require a large number of target task samples, 96 valid urban street view samples and 104 valid urban street samples were obtained in this area. The sample size distribution of the pre-trained convolutional neural network model dataset and the urban street child-friendliness evaluation model dataset is presented in the following table (Table 8).

**Table 8:** Sample size statistics for different levels of child-friendliness ratings in the Suzhou Ancient City dataset (before data augmentation).

	1 point	2 points	3 points	4 points	5 points	SUM
Pre-trained Convolutional Neural Network Model Dataset	12	14	20	29	21	96
Urban Street Child-Friendliness Evaluation Model Dataset	14	16	23	31	20	104

Field survey results show that the child-friendliness scores of urban streets in the ancient city area of Gusu District, Suzhou, are generally high. However, when the sample data of this area were input into the child-friendliness evaluation model trained on Shanghai urban samples for perception prediction, most predicted scores turned out to be low. Further field surveys on streets with score deviations identified two main types of spaces where errors occurred:

First, traditional streets with small scales and cluttered visual interfaces. The model assigned low child-friendliness scores to these streets, but local children generally perceived the small-scale architecture as providing a more intimate perspective and forming a familiar living environment, which fostered a strong sense of spatial belonging, leading them to give high scores.

Second, pedestrian spaces with dilapidated visual interfaces and no significant social activities. The model generated low child-friendliness scores for these spaces, yet local children considered them highly explorable due to dense commercial shops and well-developed small-scale lanes, thus rating them highly.

### 5.2 Transfer learning model design and evaluation

The evaluation biases mentioned above further confirm that visual cues remain a fundamental dimension of children’s assessments across different urban contexts. However, specific cultural and social-behavioral scenarios can have compensatory effects on children’s ratings, which need to be adjusted through transfer fine-tuning. Therefore, this study adopts a transfer learning strategy to fine-tune the child perceptual evaluation model. The objective is to transfer model parameters trained on samples from Shanghai’s urban area to a new target task, thereby facilitating the rapid training of an urban street child-friendliness evaluation model based on samples from the ancient city area of Gusu District in Suzhou. This approach not only reduces training costs but also addresses issues of data scarcity and overfitting in small-sample datasets. The specific steps are as follows:

First, this study loads the pre-trained weights of the convolutional neural network model trained on Shanghai data and retrains the model by inputting street view images from Suzhou along with their corresponding child-friendliness ratings. During this training process, certain convolutional layers are frozen to preserve the ability to extract abstract visual features. Only the last feature layer (layer4) and the fully connected classification layer (fc layer) are fine-tuned, allowing the model to adjust classification boundaries according to Suzhou children’s rating system.

Second, the trained convolutional neural network model is still used to extract abstract feature data from street view images. This abstract feature data is then concatenated with concrete feature data and used to train the subsequent child-friendliness evaluation model. It is important to note that, to ensure sample balance, the sample data for the Suzhou transfer model also requires data augmentation operations (Table 9). Additionally, to control data scale bias, this study maintains the Min–Max normalization range used for the Shanghai samples to verify the stability of the original model’s numerical system in the new context.

**Table 9:** Sample Size Statistics for Different Levels of Child-Friendliness Ratings in the Suzhou Ancient City dataset (after data augmentation).

	1 point	2 points	3 points	4 points	5 points	SUM
Pre-trained Convolutional Neural Network Model Dataset	60	56	60	58	63	297
Urban Street Child-Friendliness Evaluation Model Dataset	70	64	69	62	60	325

**Fig 11:**
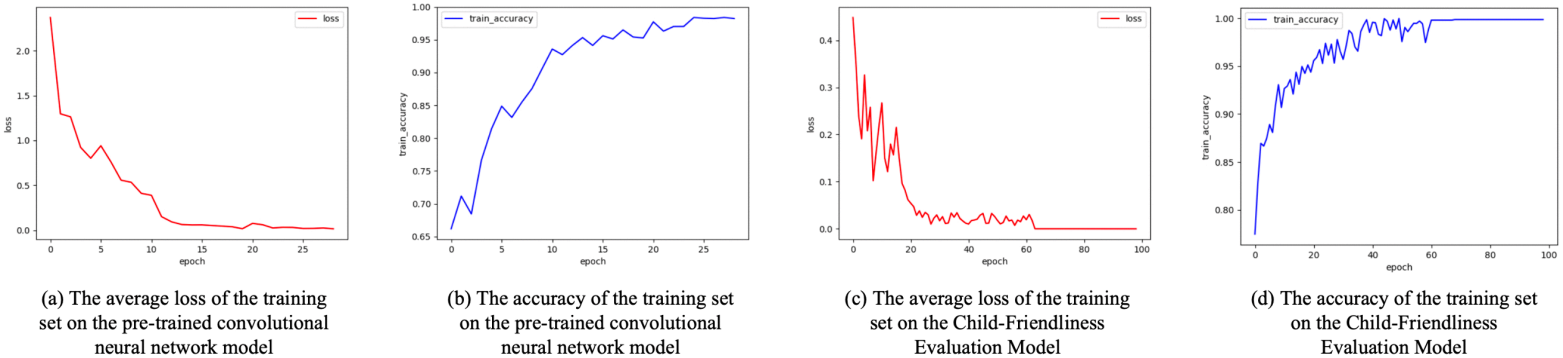
Experimental Data of the Transfer Model for Suzhou Ancient City on the Training Set. Image source: self-drawn by the author.

Considering that the sample datasets for the transfer model are all small-scale, this study divided the transfer model’s sample dataset into training and testing sets at an 8:2 ratio. Based on the experimental results from the training set (Fig 11), both the pre-trained convolutional neural network model and the urban street child-friendliness evaluation model for the Suzhou Ancient City area were able to converge rapidly. Moreover, under the same computational power, the training time for the transfer model was reduced by approximately 70% compared to the Shanghai urban area model, resulting in significantly lower time costs.

From the experimental results on the test set (Table 10), the accuracy of the transfer model slightly decreased compared to that of the original model, with deviations mainly concentrated in the transition stage between neutral scores of 3 and 4. Overall, the transfer model was still able to capture the main components of children’s evaluation patterns despite significantly different spatial structures and children’s social attributes, demonstrating that this deep learning model possesses the capability to structurally extract children’s visual and street experience judgments. It can adapt to different communities after fine-tuning with limited samples.

**Table 10:** Experimental data of the suzhou ancient city transfer model (best-performing model on the training set) on the test set.

	Pre-trained Convolutional Neural Network Model	Urban Street Child-Friendliness Evaluation Model
Accuracy	91.67%	92.31%
Recall (1 Point)	90.91%	100.00%
Precision (1Point)	100.00%	92.31%
Recall (2 Points)	91.67%	92.31%
Precision (2Points)	91.67%	100.00%
Recall (3 Points)	92.31%	92.86%
Precision (3Points)	85.71%	92.86%
Recall (4 Points)	84.62%	85.71%
Precision (4Points)	91.67%	85.71%
Recall (5 Points)	100.00%	91.67%
Precision (5Points)	91.67%	91.67%

## 6. Practical Application of the Automated Evaluation Model

### 6.1 Application of the automated evaluation model in kongjiang road subdistrict

To validate the practical application value of this urban street child-friendliness evaluation system in unassessed urban streets of Shanghai, this study selected Yangpu District’s Kongjiang Road Subdistrict as a representative case for investigation. This area was entirely independent of the model’s training, validation, and testing processes.

**Fig 12:**
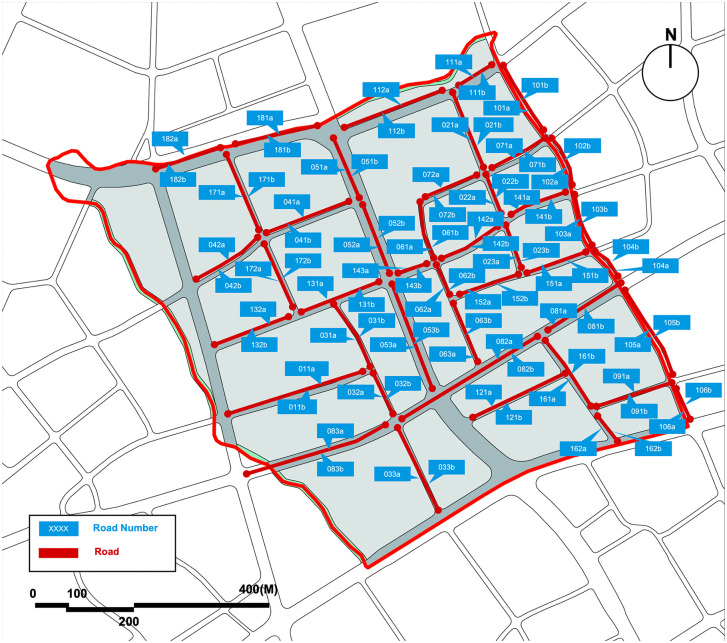
Road numbering map of Kongjiang Road Subdistrict, Yangpu District, Shanghai. Image source: self-drawn by the author. This diagram is for illustrative purposes only and may differ from the original image.).

In the Kongjiang Road Subdistrict, 43 urban streets were delineated. Following the aforementioned requirements, the concrete and abstract feature data for both sides of each street were separately recorded, resulting in a collection of 86 urban street sample datasets. These samples were then systematically numbered (Fig 12).

The practical application process of this study’s automated system in the Kongjiang Road Subdistrict of Yangpu District is as follows: First, prepare the abstract and concrete feature data of the urban streets in the area. The abstract feature data is input into a pre-trained convolutional neural network to extract urban streetscape features. Then, the extracted urban streetscape features and the concrete feature data of the urban streets are normalized using the Min-Max normalization method with fixed minimum and maximum values from the aforementioned evaluation model, rendering them dimensionless. Finally, the processed data is input into the child-friendliness evaluation model constructed in this study to generate child-friendliness scores, ultimately forming a child-friendliness evaluation matrix table (Table 11) for the urban streets in Kongjiang Road Subdistrict. This accurately depicts the perceived distribution of child-friendliness across the urban streets. In practical applications, the core objective of this system is to first identify urban streets with lower scores and then propose optimization solutions.

**Table 11:** Child-friendliness evaluation matrix of urban streets in kongjiang road subdistrict, Yangpu District, Shanghai.

Road number	Child-friendliness score	Road number	Child-friendliness score	Road number	Child-friendliness score	Road number	Child-friendliness score
011a	3	053a	2	102a	3	142a	3
011b	3	053b	3	102b	2	142b	3
021a	3	061a	3	103a	1	143a	3
021b	2	061b	2	103b	2	143b	1
022a	3	062a	3	104a	2	151a	1
022b	2	062b	3	104b	2	151b	3
023a	3	063a	2	105a	3	152a	2
023b	3	063b	3	105b	5	152b	3
031a	3	071a	3	106a	1	161a	3
031b	3	071b	3	106b	2	161b	1
032a	4	072a	3	111a	3	162a	3
032b	2	072b	2	111b	3	162b	3
033a	3	081a	3	112a	3	171a	3
033b	2	081b	3	112b	1	171b	3
041a	3	082a	3	121a	2	172a	3
041b	3	082b	3	121b	3	172b	3
042a	3	083a	4	131a	3	181a	1
042b	3	083b	5	131b	1	181b	1
051a	2	091a	1	132a	3	182a	4
051b	3	091b	2	132b	2	182b	3
052a	3	101a	3	141a	3		
052b	2	101b	3	141b	1		

**Fig 13:**
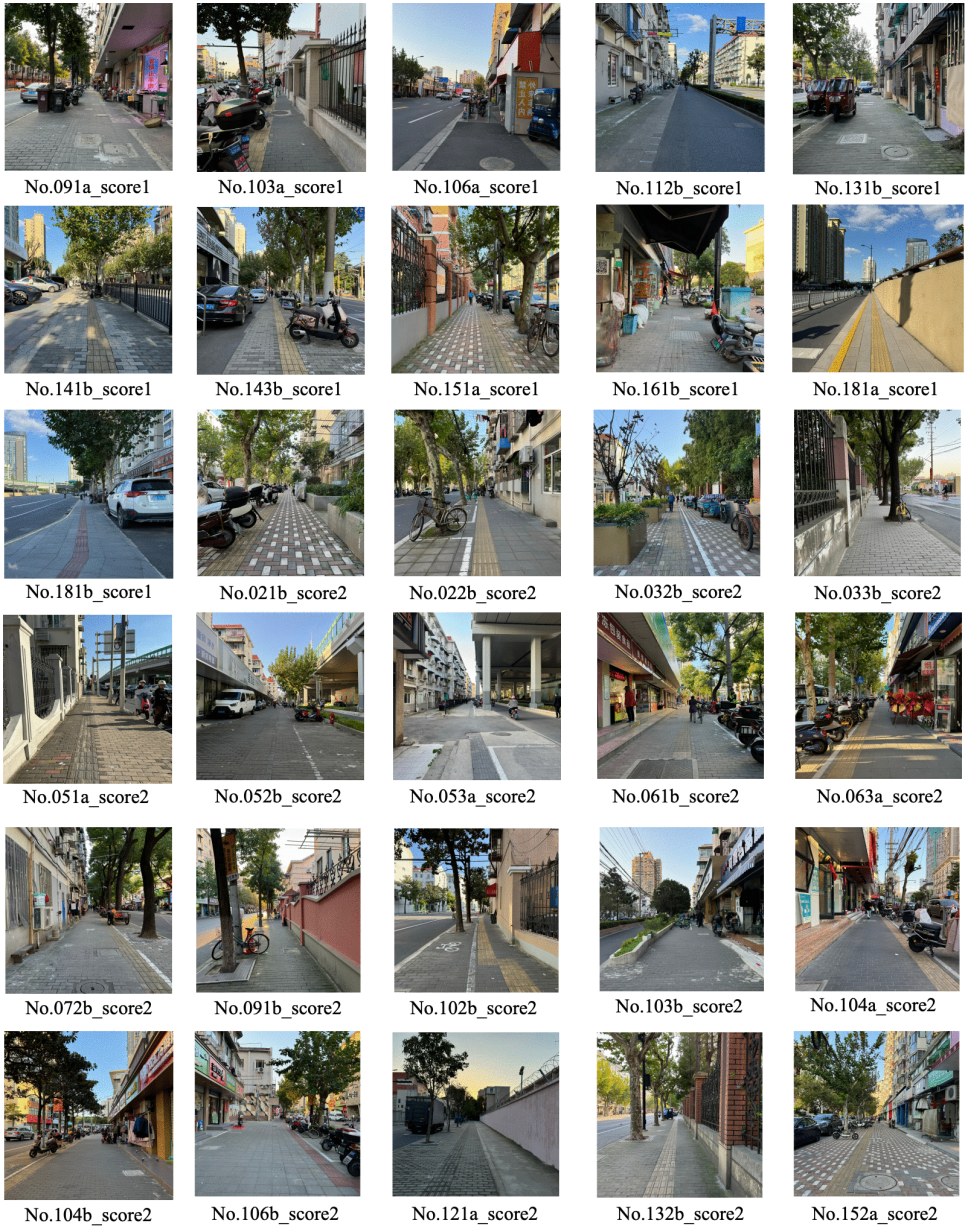
Street view images of low-rated roads in Kongjiang Road Subdistrict as predicted by the automated evaluation model. Image source: Taken by the author on site.

From the evaluation results, the child-friendliness ratings of urban streets in Kongjiang Road Subdistrict are generally low. This study focuses on 36 low-rated streets in this area with scores of 1 or 2 (Fig 13). Through offline questionnaire surveys of these low-rated streets, it was found that the evaluation results of the deep learning model are largely consistent with the survey results, with minor discrepancies in a few cases. For streets 091a and 181b, which were rated 1 by the model, the actual survey results were 2. For street 021b, which was rated 2 by the model, the actual survey result was 1, while for streets 103b and 132b, the actual survey results were 3. Field investigations and analysis of these low-rated streets reveals that the main issues are concentrated in aspects such as traffic safety hazards, poor pedestrian environment, lack of interest in space, and insufficient interactivity. Regarding traffic safety hazards, these are mainly caused by excessive traffic flow or high vehicle speeds on urban streets. Additionally, narrow sidewalks and lack of isolation facilities exacerbate traffic safety hazards, as seen in streets such as No. 112b and No. 181a. For the issue of poor pedestrian environment, almost all low-scoring streets in this area have this problem, mainly manifested in the occupation of sidewalks, severe pavement damage, and haphazard parking of motor and non-motor vehicles, with more severe issues in streets such as No. 021b, No. 022b, No. 032b, No. 103a, No. 106a, and No. 161b. Regarding the lack of interest in space, it is mainly reflected in the monotonous and dull design of urban streets, with singular visual information, single building functions, or lack of landscape, which reduces the interest of urban streets and makes it difficult to attract children to stay, such as roads No. 051a and No. 121a. As for the lack of interactivity in space, it is mainly reflected in the absence of activity spaces in urban streets where children can spontaneously explore and interact with each other, which makes children’s behavior on the streets often purely monotonous passage, lacking opportunities for interaction and communication, such as roads No. 033b, No. 053a, No. 072b, and No. 091b. Based on the specific issues identified in these low-scoring streets, urban planners and designers can propose effective urban renovation plans to promote the construction of child-friendly cities.

### 6.2 Comparison with traditional evaluation methods

**Fig 14:**
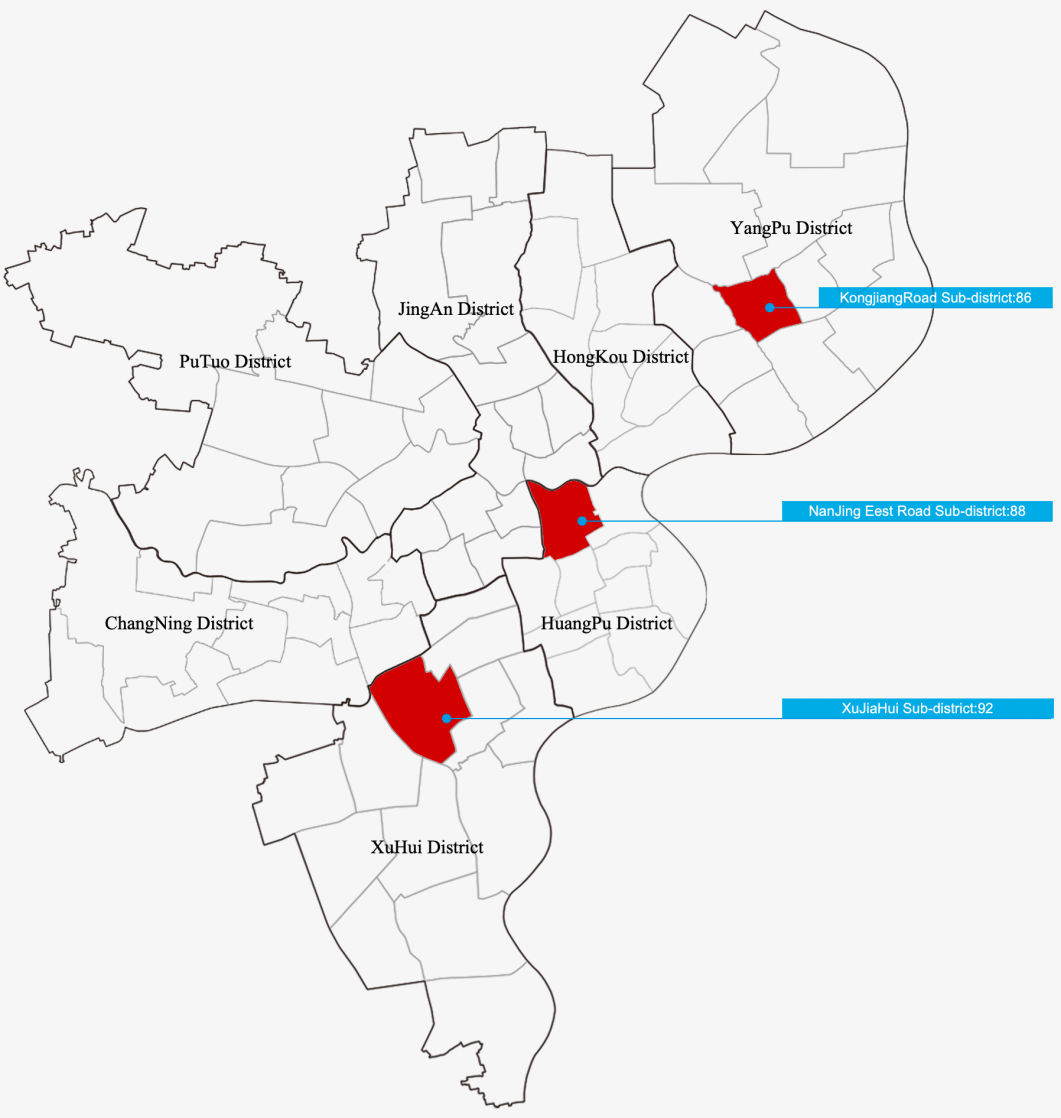
Schematic Distribution Map of Urban Street Samples in Shanghai Used for Comparative Evaluation. Image source: self-drawn by the author. This diagram is for illustrative purposes only and may differ from the original image.).

To demonstrate the efficiency and accuracy of this automated evaluation model and prove its superiority over existing evaluation tools, this study applied both the automated evaluation model and traditional child-friendliness evaluation methods to assess child-friendliness in 86 urban street samples from Kongjiang Road Subdistrict in Yangpu District, 88 urban street samples from East Nanjing Road Subdistrict in Huangpu District, and 92 urban street samples from Xujiahui Subdistrict in Xuhui District. The evaluation results from both methods were then compared with those from on-site field surveys. The urban street samples from these three areas were independent of the training, validation, and testing processes of the model in this study. Moreover, they respectively correspond to the three types of communities in Shanghai mentioned earlier: Kongjiang Road Subdistrict in Yangpu District represents a community with “old, small, and outdated” characteristics; East Nanjing Road Subdistrict in Huangpu District represents a community with modern-style characteristics; and Xujiahui Subdistrict in Xuhui District represents a community with lane-style characteristics (Fig 14).

From the evaluation process perspective, the automated evaluation model demonstrates higher efficiency. When assessing the child-friendliness of these urban street samples using the automated evaluation model, this study only needs to input street feature data into the model to obtain evaluation results within seconds. In contrast, the traditional evaluation method involves a relatively cumbersome process: first, identifying urban street features related to child-friendliness and establishing an evaluation indicator library; then, using the Analytic Hierarchy Process (AHP) to determine the weight of each street feature’s impact on child-friendliness; next, conducting questionnaire surveys to investigate children’s perceptions of each street feature’s child-friendliness and calculating the child-friendliness score for each feature using fuzzy evaluation methods; finally, multiplying each feature’s score by its corresponding weight and summing the weighted results to derive the final urban street child-friendliness rating. Among these steps, the most time- and labor-intensive tasks include establishing the evaluation indicator library, determining indicator weights using AHP, and conducting questionnaire surveys—particularly the survey process, where children in each street must evaluate the child-friendliness of all 20 street features, and this survey process must be repeated for every new urban street evaluated.

Furthermore, a critical step in the traditional evaluation method is establishing the evaluation indicator library and assigning appropriate weights to these indicators. Considering that an excessive number of feature indicators can lead to severe weight dilution when using AHP, weakening the importance of core features and distorting the weight results, this study, based on expert and children’s opinions, selected only 20 core feature indicators and assigned them corresponding weights using AHP (Table 12).

**Table 12:** Urban street feature statistics for traditional evaluation methods.

Child-Friendly Related Features	Feature Weight	Scoring Criteria (5 = Very Friendly, 4 = Relatively Friendly, 3 = Neutral, 2 = Relatively Unfriendly, 1 = Very Unfriendly)
Sidewalk Width	0.052	Whether the effective passage width on both sides of the street meets children’s safety and activity needs.
Road Surface Smoothness	0.049	Whether the road surface has damages, cracks, or other irregularities; whether the pavement material meets children’s play and stroller passage needs.
Sidewalk Continuity	0.053	Whether the sidewalk provides a continuous environment for children to walk and play.
Traffic Calming	0.042	Whether there are safety guardrails, road narrowing facilities, speed bumps or special speed zones.
Road Intersections	0.046	Whether smaller street corner radii, safety islands, and reasonable traffic signal durations are implemented.
Lighting System	0.041	Whether adequate lighting brightness is provided for children at night.
Surveillance System	0.049	Whether surveillance equipment and help-seeking devices in streets and related public spaces meet children’s safety needs.
Road Surface Comfort	0.058	Whether the road surface is smooth, slip-resistant, and has good drainage; whether elevation differences at sidewalk connections are properly handled; whether road slope settings are appropriate.
Quality of Child Facilities	0.05	Whether street furniture suitable for children’s physical scale is provided; whether child facilities are diverse and reasonably laid out.
Readability of Guidance Signs	0.045	Whether complete reminder signs aligned with children’s developmental characteristics, child-understandable crossing signs, and intelligent traffic reminders are available.
Visual Color Appropriateness	0.04	Whether the color scheme of the street space aligns with children’s preference for warm tones and rich color combinations.
Air and Acoustic Environment Quality	0.046	Whether the air quality and acoustic environment are comfortable, free from unpleasant odors or harsh noises.
Greening and Landscape Quality	0.042	Whether the quantity and quality of greenery are comfortable, meeting high green visibility and landscape diversity, and providing good shading and natural healing effects.
Public Transport Facility Accessibility	0.058	Whether the quantity and quality of bus stops within the street segment meet children’s activity radius and destination requirements, providing convenient pedestrian-to-public transport transfers.
Accessibility of Child-Friendly Spaces	0.049	Using the center of the street segment as the origin, count the number and types of bookstores, museums, children’s playgrounds, and parks within a 1000m buffer.
Street Linearity Variability	0.058	Whether the linearity of the street and sidewalk exhibits variability.
Diversity of Streetfront Shop Functions	0.057	Whether the variety and quantity of streetfront shops within the segment meet children’s daily life and learning needs (e.g., bookstores, convenience stores, stationery shops, restaurants).
Interactivity with Natural Landscapes	0.06	Whether interactive features like water scenes, mini plazas, and pocket parks are available in the street, and whether children have experienced being shooed away.
Playfulness of Street Furniture	0.052	Whether there are play facilities for children in the street; whether street furniture design meets children’s play needs.
Educational Value of Street Space	0.053	Whether the street meets children’s educational needs, featuring enlightening and inspirational planning and design.

From the evaluation results perspective, the automated evaluation model demonstrates higher accuracy. Based on the method outlined in Section 3.5 of this study, on-site questionnaire surveys were conducted to assess the child-friendliness of these urban street samples, ultimately yielding intuitive and reliable field survey evaluation results. Using these results, the credibility of the evaluation results from the two aforementioned methods can be compared (Table 13). The comparison reveals that the automated evaluation model achieves an accuracy rate of 87.22%. Among the low-rated streets, only 2 out of 21 street samples rated as 1 were mispredicted, while 4 street samples were incorrectly predicted as level 1. Similarly, only 4 out of 45 street samples rated as 2 were mispredicted, while 5 street samples were incorrectly predicted as level 2. In contrast, the traditional evaluation method achieved an accuracy rate of only 44.36%. For the low-rated streets, 13 out of 21 street samples rated as 1 were mispredicted, while 29 street samples were incorrectly predicted as level 1. Additionally, 27 out of 45 street samples rated as 2 were mispredicted, while 33 street samples were incorrectly predicted as level 2.

**Table 13:** Comparison of credibility of evaluation results between the two different evaluation methods.

	Automated evaluation model method	Traditional evaluation method
Accuracy	87.22%	44.36%
Recall (1 Point)	90.48%	38.10%
Precision (1Point)	82.61%	21.62%
Recall (2 Points)	91.11%	40.00%
Precision (2Points)	89.13%	35.29%
Recall (3 Points)	83.33%	42.22%
Precision (3Points)	89.29%	55.88%
Recall (4 Points)	87.80%	50.00%
Precision (4Points)	87.80%	56.16%
Recall (5 Points)	89.29%	46.43%
Precision (5Points)	80.65%	35.14%

Through model analysis and field research, it has been identified that the lower accuracy of the traditional evaluation method is primarily attributed to the following two factors:

First, due to the constraints of the Analytic Hierarchy Process (AHP) regarding the number of features, the traditional method only selects 20 core street features. As a result, it fails to consider a comprehensive range of street characteristics during child-friendliness evaluation. In contrast, deep learning models can retain all street features without the need for additional feature selection. Beyond extracting concrete features, deep learning models can also extract abstract features represented by street views, ensuring the completeness of street characteristics.

Second, determining weights using AHP requires constructing a judgment matrix, which involves pairwise comparisons of the importance of these 20 street features. This comparison process is subjective and limited to a small number of participants, leading to reduced credibility of the derived weights. For instance, in the traditional method used in this study, the features with the highest weight proportions included interactivity with natural landscapes, pavement comfort, accessibility of public transportation facilities, and variability in street linearity. This mechanically caused the child-friendliness ratings of all urban street samples to be disproportionately influenced by these few features. In contrast, deep learning models dynamically update weights based on loss functions. This weight determination process requires no human intervention, resulting in highly objective weight outcomes.

## 7. Discussion

### 7.1 Key factors influencing child-friendliness

In Section 4.5 above, this study employed Grad-CAM technology in the pre-trained convolutional neural network model to visually interpret key elements in street view data that influence the child-friendliness of urban streets. Additionally, SHAP technology was used in the urban street child-friendliness evaluation model to quantitatively explain the key elements in concrete feature data that affect the child-friendliness of urban streets. Based on these analytical results and field research findings, this study summarizes four key factors influencing children’s perception of street friendliness: safety, comfort, interest and interactivity.

Safety constitutes the fundamental threshold condition for children’s evaluation of street friendliness. Within the model, Abstract and concrete features such as traffic flow density, spatial boundary clarity, and proportion of visual obstructions received high feature weights among the abstract dimensions, suggesting that children’s sense of safety largely stems from perceivable order and spatial boundaries. In low-scoring samples, the CNN detected distinctive patterns—high vehicular flow, lack of separation between traffic and pedestrians, narrow sidewalks, and heavily obstructed sightlines—which closely correspond to the semantic labels “traffic” and “road” frequently mentioned in children’s questionnaires. From an environmental psychology perspective, children’s risk perception is highly dependent on the predictability of visual information: when streets lack clear boundaries or contain excessive visual noise—such as dense advertisements or overly complex façades—children tend to experience visual confusion and perceive potential threats. Therefore, the safety elements identified by the model encompass not only the physical dimension of protection, but also the cognitive dimension of controllability.

Comfort represents the next essential dimension for children’s positive evaluation of street friendliness once safety is ensured. The model indicates that concrete features such as sidewalk width, green coverage rate, and number of facilities are strongly correlated with high-rated streets. In terms of visual abstraction, street-view images exhibiting bright colors, spatial openness, and continuous tree canopies elicited strong activations in the network’s feature extraction layers. Children’s sense of comfort is derived from two categories of factors: physiological adaptation and psychological stability. The former relates to environmental conditions such as temperature, shade, and air circulation, while the latter is associated with spatial openness, continuity, and order. In high-rated streets, the model prominently activated areas characterized by greenery, light-toned surfaces, coherent pedestrian paths, and unobstructed backgrounds, whereas in low-rated streets, the model responded to cluttered, dense, and dim visual regions. This demonstrates that the deep learning model captured not only the visual correlates of physical comfort, but also perceptual cues linked to psychological comfort.

Interest serves as the intrinsic motivational factor driving children’s active engagement with street spaces. Model analysis reveals that color saturation, façade diversity, and visual complexity of street elements are significantly and positively associated with high child-friendliness scores. Specifically, among the concrete features, abundant landscapes and diverse building functions—particularly a high proportion of commercial buildings—are more favored by children. Deeper layers of the ResNet18 network showed the strongest activations for images with high color contrast, varied morphological compositions, and decorative details, indicating that the model effectively identified “visual richness,” a core attribute of children’s visual preferences. Field observations further support that interest extends beyond an aesthetic response; it functions as a behavioral catalyst. Streets with high visual diversity and exploratory potential encourage longer dwell times and more varied activities among children. Open-ended questionnaire responses such as “this street is fun to play on” reveal that playfulness stimulates exploratory motivation. Thus, interest can be conceptualized as a perception–behavior feedback loop: visual stimuli attract attention, attention drives exploratory behavior, and positive exploration reinforces spatial memory and sense of belonging.

Interactivity represents the most socially oriented dimension of children’s street perception, reflecting the publicness and openness of streets as social spaces. The model’s analysis shows that streets featuring open plazas, corner seating, low fences, and multiple access points achieved significantly higher predicted scores. These elements manifested visually as cues of high spatial permeability, distinct activity nodes, and visible clusters of people or objects. Questionnaire results further indicate that children preferred streets where they could play and interact with peers, suggesting that their spatial understanding extends beyond individual safety or comfort to encompass opportunities for social interaction. Notably, interactivity also correlates with street-network connectivity. The model observed that streets situated near multifunctional nodes such as schools, parks, and commercial areas tended to receive higher scores, implying that children’s perception of streets is shaped not only by local spatial features but also by broader factors such as path continuity and socio-functional diversity.

### 7.2 Practical recommendations for child-friendly streets

Based on the results of the deep learning model and field validation, this study identifies the primary mechanisms influencing the child-friendliness of urban streets and proposes targeted spatial optimization strategies accordingly.

In terms of safety, the model identified distinct features in low-scoring street samples, including high motor vehicle density, a lack of separation facilities, and high levels of visual disorder. Accordingly, a comprehensive safety system can be constructed from three perspectives: visual safety, behavioral guidance, and dynamic protection. First, spatial legibility can be enhanced through high-contrast pavement materials, low-level warning lights, and child-height signage systems to help children quickly understand traffic flow and risk boundaries [33]. Second, low hedges, flexible buffer strips, and semi-transparent guardrails can be installed at street nodes to create “soft boundaries” that establish psychological buffers while preserving children’s freedom to explore. Finally, a street safety monitoring platform can be developed based on the deep learning model, integrating real-time traffic video and pedestrian detection technologies to automatically identify and alert high-risk nodes, thereby providing continuous intelligent protection for children’s travel.

In terms of comfort, the model results show that streets with low green coverage, damaged sidewalks, and inadequate lighting received lower scores, while high-scoring streets generally exhibit visual characteristics of uniform illumination, abundant vegetation, and spatial openness. Therefore, comfort optimization can be approached from two aspects: microclimate regulation and scale adaptation. First, continuous tree canopies and shaded corridors should be created on both sides of sidewalks, with permeable pavements and localized evaporative cooling devices used to alleviate thermal stress. Smart lighting systems should be installed in under-illuminated areas to enhance nighttime safety and visibility. Second, to address the problem of narrow pedestrian spaces identified by the model, the proportion between sidewalks and non-motorized lanes should be adjusted, and temporary rest nodes and seating facilities should be added [34]. Child-scale wayfinding systems should be arranged at intersections so that spatial signage better corresponds to children’s perceptual height and cognitive range.

At the level of interest, the deep learning model identified low-scoring features such as “monotonous colors,” “repetitive façades,” and “insufficient landscape decoration,” whereas streets with higher child-friendliness typically demonstrated greater richness in color saturation, morphological diversity, and spatial layering. Optimization strategies can therefore be developed from two perspectives: multi-sensory design and exploration-oriented space. First, interactive paving, light-and-shadow installations, and façade patterns can be introduced as visual and tactile elements to stimulate children’s curiosity and exploratory behavior. Pavement surfaces can incorporate hopscotch grids, puzzle paths, or animal footprints to transform streets into gamified experiential environments [35]. Second, deep learning models can be used to identify low-sensory-stimulation zones across the city, into which storytelling-themed trails and community art installations can be introduced to reinforce the cultural narrative of the street, enabling children to achieve both cognitive and emotional engagement during movement.

In terms of interactivity, the model revealed that high-scoring streets commonly exhibit characteristics such as “distinct open nodes,” “high public seating density,” and “strong visual connectivity,” indicating that children’s interactive experiences are closely related to spatial dwellability and social potential. Accordingly, interactivity optimization can be systematically advanced through interventions at the node, path, and community levels. First, additional seating, reading corners, and sliding rails can be installed at street corners and pocket plazas to create composite urban furnishings that function as “social touchpoints.” Second, residential areas, schools, and parks—key spaces frequently used by children—should be connected through pedestrian-priority corridors to form a “continuous and accessible child-friendly network,” thereby reducing traffic-related barriers to children’s mobility [2]. Finally, children should be invited to participate in the co-creation of street graphics, facility naming, and behavioral rules, transforming their role from “users” to “co-creators” in the process of urban renewal, thereby strengthening their sense of social belonging and responsibility.

### 7.3 Limitations and future directions

Although this study achieves an automated evaluation of child-friendliness in urban streets through a deep learning model that integrates abstract and concrete features, certain limitations remain.

First, the core inputs of the current model are street view images and structural street data, which primarily reflect children’s perceptual dimensions of the visual environment. However, children’s actual experiences of streets are multisensory, involving auditory (noise), olfactory (smell), somatic (temperature and humidity), and physiological (heart rate and electrodermal response) dimensions. While deep learning models can effectively simulate visual perception, they have limited capacity to directly capture these embodied and situational experiences. Future research could integrate soundscape data (e.g., spatial distribution of traffic and natural sounds), meteorological and microclimate monitoring data (temperature, humidity, illumination intensity), and physiological or affective signals collected via wearable devices, such as heart rate and electrodermal activity, to construct a multimodal deep learning framework capable of depicting children’s bodily and emotional responses.

Second, the questionnaire respondents in this study were children aged 7–12, primarily from urban areas. While this group is representative in terms of travel independence, educational background, and living environment, it does not include samples from children with disabilities. Their sense of security, spatial usage, and psychological needs regarding streets may differ significantly from those of non-disabled children. Additionally, although this study included areas such as “workers’ new villages” to cover low-income and migrant worker families, privacy protection constraints during the questionnaire survey prevented the collection of specific labels such as household income or household registration status of the participating children. As a result, detailed group difference analyses could not be conducted, potentially obscuring the special needs of children from marginalized backgrounds. Future research should establish stratified sampling mechanisms to specifically target children with disabilities and well-defined low-income groups, thereby building a more inclusive perceptual database.

Third, the training samples for the evaluation model constructed in this study were entirely sourced from urban Shanghai, with no data from other medium- or small-sized cities or cities with different cultural backgrounds included in the training. Although transfer learning was applied to adapt the model to the ancient city area of Suzhou, and fine-tuning validated its robustness, this result remains a localized validation. It does not yet demonstrate that the proposed method for constructing a child-friendliness evaluation system for urban streets is universally applicable. Therefore, future research should expand its scope by incorporating more diverse urban samples, such as those from medium- and small-sized cities, suburban streets, and areas with distinct cultural characteristics. This would help establish a comprehensive cross-context framework, enhancing the stability and transferability of visual features, concrete indicators, and children’s judgment boundaries across multiple scenarios.

## 8. Conclusion

Using Shanghai’s urban streets as a case study, this study constructed a deep learning–based evaluation model integrating abstract and concrete features by combining street view imagery, spatial structural characteristics, and children’s subjective perception data, thereby achieving an automated assessment of child-friendliness in urban streets. The results demonstrate that the proposed model can accurately identify varying levels of child-friendliness across different street samples and reveal four key dimensions influencing children’s perceptual experience: safety, comfort, playfulness, and interactivity.

The contributions of this study are as follows: (1) By employing deep neural networks as the core analytical framework, it establishes a quantitative model that maps multidimensional urban information to children’s perceptual responses, thereby significantly enhancing the scientific rigor, objectivity, and operational efficiency of urban street evaluations. (2) It proposes an algorithmic framework capable of fusing abstract and concrete features of urban streets, which demonstrates strong practical applicability and can be extended to multidimensional feature extraction in other urban contexts. (3) Based on model validation, the study identifies four core dimensions affecting child-friendliness and proposes data-driven spatial optimization strategies for creating more child-friendly urban streets.

## Supporting information

S1 Code The Code of Child-Friendly Evaluation Model. (PDF)

## References

[1] Tayefi Nasrabadi M, García EH, Pourzakarya M. Let children plan neighborhoods for a sustainable future: a sustainable child-friendly city approach. Local Environment. 2021;26(2):198–215. doi: 10.1080/13549839.2021.1884668

[2] Zhao J, Su W, Luo J, Zuo J. Evaluation and optimization of walkability of children’s school travel road for accessibility and safety improvement. Int J Environ Res Public Health. 2021;19(1):71. doi: 10.3390/ijerph19010071 35010339 PMC8751143

[3] Rigolon A, Toker Z, Gasparian N. Who has more walkable routes to parks? An environmental justice study of Safe Routes to Parks in neighborhoods of Los Angeles. Journal of Urban Affairs. 2017;40(4):576–91. doi: 10.1080/07352166.2017.1360740

[4] Brussoni M, Lin Y, Han C, Janssen I, Schuurman N, Boyes R, et al. A qualitative investigation of unsupervised outdoor activities for 10- to 13-year-old children: “I like adventuring but I don’t like adventuring without being careful”. Journal of Environmental Psychology. 2020;70:101460. doi: 10.1016/j.jenvp.2020.101460

[5] DHaese S, Vanwolleghem G, Hinckson E, De Bourdeaudhuij I, Deforche B, Van Dyck D, et al. Cross-continental comparison of the association between the physical environment and active transportation in children: a systematic review. Int J Behav Nutr Phys Act. 2015;12:145. doi: 10.1186/s12966-015-0308-z 26610344 PMC4660808

[6] Ramezani S, Said I. Children’s nomination of friendly places in an urban neighbourhood in Shiraz, Iran. Children’s Geographies. 2012;11(1):7–27. doi: 10.1080/14733285.2012.742699

[7] Murota M. A study on the use of parks in the green matrix system of Kohoku New Town, Japan. Journal of Asian Architecture and Building Engineering. 2009;8(1):73–9. doi: 10.3130/jaabe.8.73

[8] Kruse J, Kang Y, Liu Y-N, Zhang F, Gao S. Places for play: Understanding human perception of playability in cities using street view images and deep learning. Computers, Environment and Urban Systems. 2021;90:101693. doi: 10.1016/j.compenvurbsys.2021.101693

[9] Wu Y, Liu Q, Hang T, Yang Y, Wang Y, Cao L. Integrating restorative perception into urban street planning: A framework using street view images, deep learning, and space syntax. Cities. 2024;147:104791. doi: 10.1016/j.cities.2024.104791

[10] Pitsikali A, Parnell R, McIntyre L. The public value of child-friendly space. ARCH. 2020;14(2):149–65. doi: 10.1108/arch-07-2019-0164

[11] Torres J, Cloutier M-S, Bergeron J, St-Denis A. ‘They installed a speed bump’: children’s perceptions of traffic-calming measures around elementary schools. Children’s Geographies. 2019;18(4):477–89. doi: 10.1080/14733285.2019.1685075

[12] von Stülpnagel R, Riach N, Hologa R, Kees J, Gössling S. School route safety perceptions of primary school children and their parents: Effects of transportation mode and infrastructure. International Journal of Sustainable Transportation. 2024;18(6):465–77. doi: 10.1080/15568318.2024.2350992

[13] Wang X, Huang J, Qin Z, Gan W, He Z, Li X. Is the children’s 15-minute city an effective framework for enhancing children’s health and well-being? an empirical analysis from Western China. Buildings. 2025;15(2):248. doi: 10.3390/buildings15020248

[14] Wang R, Liu J. Research on ecological-cultural coupling evaluation and optimization strategy of urban ecological parks: A case study of Yubei District, Chongqing. Journal of Human Settlements in West China. 2024;39(4):157–64.

[15] Jin S, Deng Y, Tu H. Reprogramming Urban Communities: Enhancing Children’s Well-being through a Value-Based Programming Approach in Inclusive Planning and Design. In: Proceedings of the 60th ISOCARP World Planning Congress. 2024. doi: 10.47472/c6quucj8

[16] Moore KA. Developing an indicator system to measure child well-being: lessons learned over time. Child Ind Res. 2019;13(2):729–39. doi: 10.1007/s12187-019-09644-4

[17] Jiang S, Wang L, Cheng Y. Unrevealing the mediating mechanisms between material deprivation and children’s life satisfaction: empirical evidence from the international survey of children’s well-being. Applied Research Quality Life. 2022;18(2):893–914. doi: 10.1007/s11482-022-10101-8

[18] Sheng K, Liu L, Wang F, Li S, Zhou X. An eye-tracking study on exploring children’s visual attention to streetscape elements. Buildings. 2025;15(4):605. doi: 10.3390/buildings15040605

[19] Zhang R, Huang Q, Peng Z, Zhang X, Shang L, Yang C. Evaluating the impact of elementary school urban neighborhood color on children’s mentalization of emotions through multi-source data. Buildings. 2024;14(10):3128. doi: 10.3390/buildings14103128

[20] Talaei Khoei T, Ould Slimane H, Kaabouch N. Deep learning: systematic review, models, challenges, and research directions. Neural Comput & Applic. 2023;35(31):23103–24. doi: 10.1007/s00521-023-08957-4

[21] Li F, Yigitcanlar T, Nepal M, Nguyen K, Dur F. Machine learning and remote sensing integration for leveraging urban sustainability: A review and framework. Sustainable Cities and Society. 2023;96:104653. doi: 10.1016/j.scs.2023.104653

[22] Baduge SK, Thilakarathna S, Perera JS, Arashpour M, Sharafi P, Teodosio B, et al. Artificial intelligence and smart vision for building and construction 4.0: Machine and deep learning methods and applications. Automation in Construction. 2022;141:104440. doi: 10.1016/j.autcon.2022.104440

[23] Mo Y, Wu Y, Yang X, Liu F, Liao Y. Review the state-of-the-art technologies of semantic segmentation based on deep learning. Neurocomputing. 2022;493:626–46. doi: 10.1016/j.neucom.2022.01.005

[24] Huang Y, Zhang F, Gao Y, Tu W, Duarte F, Ratti C, et al. Comprehensive urban space representation with varying numbers of street-level images. Computers, Environment and Urban Systems. 2023;106:102043. doi: 10.1016/j.compenvurbsys.2023.102043

[25] Ghanbari M, Karimi M, Claramunt C, Lagesse C. Enhancing urban transportation flow modeling through a graph neural network-based spatially weighted interaction model: a case study of chicago taxi data. J geovis spat anal. 2025;9(1). doi: 10.1007/s41651-025-00217-4

[26] Tang F, Zeng P, Wang L, Zhang L, Xu W. Urban perception evaluation and street refinement governance supported by street view visual elements analysis. Remote Sensing. 2024;16(19):3661. doi: 10.3390/rs16193661

[27] Yao Y, Liang Z, Yuan Z, Liu P, Bie Y, Zhang J, et al. A human-machine adversarial scoring framework for urban perception assessment using street-view images. International Journal of Geographical Information Science. 2019;33(12):2363–84. doi: 10.1080/13658816.2019.1643024

[28] Yang Y, Wang Q, Wu D, Hang T, Ding H, Wu Y, et al. Constructing child-friendly cities: Comprehensive evaluation of street-level child-friendliness using the method of empathy-based stories, street view images, and deep learning. Cities. 2024;154:105385. doi: 10.1016/j.cities.2024.105385

[29] Tu H, Miao X, Jin S, Yang J, Miao X, Qi J. Deep learning-based systems for evaluating and enhancing child-friendliness of Urban Streets—A case of shanghai Urban Street. Buildings. 2025;15(13):2291. doi: 10.3390/buildings15132291

[30] Ye Y, Zeng W, Shen Q, Zhang X, Lu Y. The visual quality of streets: A human-centred continuous measurement based on machine learning algorithms and street view images. Environment and Planning B: Urban Analytics and City Science. 2019;46(8):1439–57. doi: 10.1177/2399808319828734

[31] Shafik N, Mansour Y, Kamel S, Morcos R. The impact of the cairo streets development project on the independent mobility of children: a field study on the streets of Heliopolis, Egypt. Infrastructures. 2021;6(7):98. doi: 10.3390/infrastructures6070098

[32] Child Height Status Report. CCTF; 2024. https://www.cctf.org.cn/d/file/report/study/2024-09-12/ddc996ecaca57bc89b85069cc40d2761.pdf

[33] Kweon B-S, Shin W-H, Ellis CD. School walk zone: identifying environments that foster walking and biking to school. Sustainability. 2023;15(4):2912. doi: 10.3390/su15042912

[34] Haotian H, Jing H, Xinfeng J, Jiantao Z, Jianwei G. Evaluation of route choice for walking commutes to school and street space optimization in old urban areas of China based on a child‐friendly orientation: The case of the Wuyi Park area in Zhengzhou. Children & Society. 2023;38(5):1527–56. doi: 10.1111/chso.12817

[35] Lee WM, Park HS, Kim SN, Kim JC, Lee KH. Effects of elementary school neighbourhood environment on children’s play activities: a case study of GaeMyong elementary school neighbourhood. International Journal of Urban Sciences. 2019;24(1):88–109. doi: 10.1080/12265934.2019.1570862

