# Supplementary material for: A deep learning-based evaluation system for child-friendly urban streets integrating abstract and concrete features—A case of Shanghai Urban Street
Source: PLoS One. 2026 Feb 23;21(2):e0342430. doi: 10.1371/journal.pone.0342430 (PMC12928418; doi:10.1371/journal.pone.0342430)
Supplement: S1 Code — (PDF) [file pone.0342430.s001.pdf]

```
import pandas as pd
from sklearn.model_selection import train_test_split
from sklearn.model_selection import StratifiedKFold
import numpy as np
import torch
import torch.nn as nn
import torch.optim as optim
from torch.utils.data import TensorDataset, DataLoader
import time
from sklearn.preprocessing import MinMaxScaler
import csv
import warnings
import os


os.makedirs("./model", exist_ok=True)
os.makedirs("./data", exist_ok=True)
warnings.filterwarnings('ignore')


def create_dataset():
    file_path = "./street_features_all.xlsx"
    data_frame = pd.read_excel(file_path)
    data = data_frame.values

    x = data[:, :-1].astype(np.float32)
    y = data[:, -1].astype(np.int64)

    x_temp, x_test, y_temp, y_test = train_test_split( x, y, test_size=0.2,
random_state=66, stratify=y)

    transfer = MinMaxScaler()
    x_temp = transfer.fit_transform(x_temp)
    x_test = transfer.transform(x_test)

    dataset_test = TensorDataset(torch.from_numpy(x_test),
torch.from_numpy(y_test))
    dataloader_test = DataLoader(dataset_test, batch_size=1, shuffle=False)

    return x_temp, y_temp, x_test, y_test, dataloader_test, transfer


class EvaluationModel(nn.Module):
    def __init__(self, input_dim, output_dim):
```

```
        super(EvaluationModel, self).__init__()
        self.linear1 = nn.Linear(input_dim, 512)
        self.linear2 = nn.Linear(1024, 2048)
        self.linear3 = nn.Linear(2048, 4096)
        self.linear4 = nn.Linear(4096, output_dim)

    def forward(self, x1, x2):
        x = torch.relu(self.linear1(x1))
        x = torch.cat((x, x2), dim=1)
        x = torch.relu(self.linear2(x))
        x = torch.relu(self.linear3(x))
        x = self.linear4(x)
        return x


def val_evaluate(val_dataloader, model):
    val_total = 0
    val_correct = 0
    model.eval()
    with torch.no_grad():
        for x, targets in val_dataloader:
            outputs = model(x[:, :50], x[:, 50:])
            predicts = torch.argmax(outputs, dim=1)
            val_total += targets.size(0)
            val_correct += (predicts == targets).sum().item()
    return val_correct / val_total


def k_fold_train():
    x_temp, y_temp, x_test, y_test, dataloader_test, transfer = create_dataset()

    kf = StratifiedKFold(n_splits=5, shuffle=True, random_state=66)
    fold_results = []

    for fold, (train_idx, val_idx) in enumerate(kf.split(x_temp, y_temp), 1):
        print(f"\n===== Fold {fold} Cross-Validation =====")

        x_train, x_val = x_temp[train_idx], x_temp[val_idx]
        y_train, y_val = y_temp[train_idx], y_temp[val_idx]

        dataset_train = TensorDataset(torch.from_numpy(x_train),
torch.from_numpy(y_train))
        dataset_val = TensorDataset(torch.from_numpy(x_val),
torch.from_numpy(y_val))
```

```
dataloader_train = DataLoader(dataset_train, batch_size=32, shuffle=True)
dataloader_val = DataLoader(dataset_val, batch_size=32, shuffle=False)

model = EvaluationModel(50, 5)    # 输入维度 50，输出维度 5（类别数）
criterion = nn.CrossEntropyLoss()
optimizer = optim.Adam(model.parameters(), lr=1e-3)

fold_train_loss = []
fold_train_acc = []
fold_val_acc = []
best_val_acc = 0.0
best_model_path = f"./model/evaluation_fold{fold}_best.bin"

for epoch in range(100):
    start_time = time.time()
    model.train()
    total_loss = 0.0
    correct = 0
    total = 0

    for x, y in dataloader_train:
        optimizer.zero_grad()
        outputs = model(x[:, :50], x[:, 50:])
        loss = criterion(outputs, y)
        loss.backward()
        optimizer.step()

        total_loss += loss.item() * x.size(0)
        _, predicted = torch.max(outputs.data, 1)
        total += y.size(0)
        correct += (predicted == y).sum().item()

    train_loss = total_loss / total
    fold_train_loss.append(train_loss)

    train_acc = correct / total
    fold_train_acc.append(train_acc)

    val_acc = val_evaluate(dataloader_val, model)
    fold_val_acc.append(val_acc)

    if val_acc > best_val_acc:
        best_val_acc = val_acc
```

```
                torch.save(model.state_dict(), best_model_path)

            epoch_time = time.time() - start_time
            print(f"Fold {fold} Epoch {epoch + 1}/100 - Loss: {train_loss:.4f}, Train
Accuracy: {train_acc:.4f}, "
                  f"Val Accuracy: {val_acc:.4f}, Time: {epoch_time:.2f}s")


        model.load_state_dict(torch.load(best_model_path))
        test_acc = val_evaluate(dataloader_test, model)
        fold_results.append({
            "fold": fold,
            "best_val_acc": best_val_acc,
            "test_acc": test_acc
        })
        print(f"Fold {fold} Best Validation Accuracy: {best_val_acc:.4f}, Test
Accuracy: {test_acc:.4f}")

        with open(f"./data/fold{fold}_train_loss.csv", "w", newline="") as f:
            writer = csv.writer(f)
            for loss in fold_train_loss:
                writer.writerow([loss])
        with open(f"./data/fold{fold}_train_acc.csv", "w", newline="") as f:
            writer = csv.writer(f)
            for acc in fold_train_acc:
                writer.writerow([acc])
        with open(f"./data/fold{fold}_val_acc.csv", "w", newline="") as f:
            writer = csv.writer(f)
            for acc in fold_val_acc:
                writer.writerow([acc])

    print("\n===== 5-Fold Cross-Validation Summary =====")
    val_accs = [r["best_val_acc"] for r in fold_results]
    test_accs = [r["test_acc"] for r in fold_results]
    print(f"Average Validation Accuracy: {np.mean(val_accs):.4f} ±
{np.std(val_accs):.4f}")
    print(f"Average Test Accuracy: {np.mean(test_accs):.4f} ±
{np.std(test_accs):.4f}")

    with open("./data/cross_validation_results.csv", "w", newline="") as f:
        writer = csv.writer(f)
        writer.writerow(["fold", "best_val_acc", "test_acc"])
        for r in fold_results:
            writer.writerow([r["fold"], r["best_val_acc"], r["test_acc"]])
```

```python
def my_test():
    _, _, _, _, dataloader_test, _ = create_dataset()
    model = EvaluationModel(50, 5)

    best_model_path = "./model/evaluation_fold1_best.bin"
    model.load_state_dict(torch.load(best_model_path))

    test_acc = val_evaluate(dataloader_test, model)
    print(f"Final Test Set Accuracy: {test_acc:.4f}")


if __name__ == '__main__':
    k_fold_train()
    # my_test()
```
